# Long-term study of behaviors of two cohabiting sea urchin species, *Mesocentrotus nudus* and *Strongylocentrotus intermedius*, under conditions of high food quantity and predation risk in situ

**DOI:** 10.7717/peerj.8087

**Published:** 2019-11-22

**Authors:** Peter M. Zhadan, Marina A. Vaschenko

**Affiliations:** 1 Department of Geochemistry and Ecology of the Ocean, V. I. Il’ichev Pacific Oceanological Institute FEB RAS, Vladivostok, Russia; 2 Laboratory of Physiology, A.V. Zhirmunsky National Scientific Center of Marine Biology FEB RAS, Vladivostok, Russia

**Keywords:** Predator–prey interactions, Sea urchin behavior, Escape response, Alarm response duration, Grouping behavior, Chemical alarm cues, Defensive adaptation

## Abstract

**Background:**

In the predator–sea urchin–macrophyte trophic cascade, the ecological effect of sea urchins as grazers depends both on their density and the changes in foraging activity, which are influenced by various disturbing factors. However, the complete duration of the alarm reactions of echinoids has not been studied until now. Here, we tested a hypothesis that two cohabiting sea urchins, *Mesocentrotus nudus* and *Strongylocentrotus intermedius*, which differ morphologically, might display different behavioral responses to high hydrodynamic activity and predation.

**Methods:**

We used continuous time-lapse video recording to clarify behavioral patterns of *M. nudus* and *S. intermedius* in presence of a large quantity of food (the kelp *Saccharina japonica*) but under different weather conditions and different types of predation threat: (1) calm weather conditions, (2) stormy weather conditions, (3) predation risk associated with the presence of several sea star species and (4) predation risk associated with an alarm stimulus (crushed conspecifics or heterospecifics). Three separate video recording experiments (134 days in total) were conducted under field conditions. Video recording analysis was performed to determine the number of specimens of each sea urchin species in the cameras’ field of view, size of sea urchins’ groups, movement patterns and the duration of the alarm responses of both sea urchin species.

**Results:**

We showed that in the presence of kelp, *M. nudus* and *S. intermedius* exhibited both similar and different behavioral responses to hydrodynamics and predation threat. Under calm weather, movement patterns of both echinoids were similar but *M. nudus* exhibited the higher locomotion speed and distance traveled. Furthermore, *S. intermedius* but not *M. nudus* tended to group near the food substrate. The stormy weather caused a sharp decrease in movement activity followed by escape response in both echinoids. Six starfish species failed to predate on healthy sea urchins of either species and only a few attacks on ailing *S. intermedius* specimens were successful. The alarm response of *S. intermedius* lasted approximately 90 h and 20 h for starfish attacks on ailing conspecifics and for simulated attacks (crushed conspecifics or heterospecifics), respectively and involved several phases: (1) flight response, (2) grouping close to the food, (3) leaving the food and (4) return to the food. Phase three was the more pronounced in a case of starfish attack. *M. nudus* only responded to crushed conspecifics and exhibited no grouping behavior but displayed fast escape (during 4 h) and prolonged (up to 19 days) avoidance of the food source. This outcome is the longest alarm response reported for sea urchins.

**Discussion:**

The most interesting finding is that two cohabiting sea urchin species, *M. nudus* and *S. intermedius*, display different alarm responses to predation threat. Both alarm responses are interpreted as defensive adaptations against visual predators.

## Introduction

The need to forage and the need to avoid predation are considered as the most important evolutionary forces in the selection of morphological and behavioral characteristics of animals (Lima, 1998; Lima & Dill, 1990). Sea urchins (class Echinoidea), which have a long evolutionary history and a worldwide distribution, have developed a number of defense mechanisms against predators promoting survival and reproduction. Possessing a locomotion speed that is significantly inferior to that of many potential predators, sea urchins have a hard, internal calcium carbonate skeleton (test) covered by spines. Some species are additionally protected by pedicellaria with poison glands (Jensen, 1966; Cannone, 1970). Sea urchins exhibit mostly nocturnal activity for both the movement/migration (Crook, Long & Barnes, 2000; Dance, 1987; Hasegawa, 2014; Hereu et al., 2005) and spawning (Zhadan, Vaschenko & Ryazanov, 2019), appearing to be an adaptation for enemy avoidance (Fricke, 1973).

Sea urchins lack image-forming eyes (Ullrich-Lüter et al., 2011); therefore, chemical senses are an essential source of information on predation risk. The emergence of chemical cues (substances emitted by predators and/or physically damaged con- or heterospecific prey) can cause various behavioral reactions in sea urchins: “cryptic behavior”, that is, hiding in a crack or hole (Fricke, 1973; Kintzing & Butler, 2014a; Spyksma, Taylor & Shears, 2017); “associative behavior”, that is, aggregation into dense groups (Bernstein, Schroeter & Mann, 1983; Hagen, Andersen & Stabell, 2002; Kintzing & Butler, 2014a), and “dispersion behavior”, that is, escape (Hagen, Andersen & Stabell, 2002; Urriago, Himmelman & Gaymer, 2011; Vadas & Elner, 2003). For some species, it has been shown that the emergence of a chemical signal from predator presence was associated with a decrease in the intensity of feeding (the so-called “fear effect”) (Freeman, 2006; Kintzing & Butler, 2014b; Matassa, 2010; Spyksma, Taylor & Shears, 2017). The changes in behavior, including a decrease in foraging activity, were more pronounced in young individuals (Clemente et al., 2013; Freeman, 2006), and these behaviors may not occur in large well-armed adults (Parker & Shulman, 1986; Wirtz & Duarte, 2012).

Being consumers of macrophytes, sea urchins severely affect the structure of coastal benthic communities (see for review Estes & Duggins, 1995; Mann, 1982; Steneck, 2013). There is growing evidence that in the predator–prey–plant trophic cascade, the ecological effect can be not only prey density-mediated (i.e., associated with the direct influence of predators on prey number) but also prey behavior-mediated (i.e., associated with inhibition of prey foraging activity by predators) (Abrams, 1995; Dill, Heithaus & Walters, 2003; Pearson, 2010; Schmitz, Krivan & Ovadia, 2004; Trussell, Ewanchuk & Matassa, 2006; Werner & Peacor, 2003), prompting the suggestion that the grazing effect of sea urchins on macrophytes’ abundance may depend on the duration of their alarm response. However, the complete duration of the alarm responses of sea urchin species to waterborne chemical signals emitted either from other prey (conspecific or non-conspecific), the predator itself, or both has remained unknown until now. Most studies of sea urchins’ responses to chemical alarm signals have been conducted under laboratory conditions (Chivers & Smith, 1998; Hagen, Andersen & Stabell, 2002; Kintzing & Butler, 2014a, 2014b; Manzur & Navarrete, 2011; Matassa, 2010; Spyksma, Taylor & Shears, 2017) and field experiments conducted under conditions of calm water and under unidirectional water flows (Parker & Shulman, 1986; Snyder & Snyder, 1970; Vadas & Elner, 2003; Wirtz & Duarte, 2012; but see Manzur & Navarrete, 2011; Urriago, Himmelman & Gaymer, 2011). All these studies have focused on the initial stage of sea urchins’ alarm response, whereas the entire sequence of behavioral events in sea urchins in their natural habitat, starting from the onset of the alarm reaction to its complete extinction, has not been investigated.

The present study was undertaken to identify the temporal and spatial parameters of the complete alarm reactions in two species of sea urchins, *Strongylocentrotus intermedius* (A. Agassiz, 1864) and *Mesocentrotus nudus* (A. Agassiz, 1864) (= *Strongylocentrotus nudus*) under conditions of food abundance using continuous time-lapse video recording (during 1.5–2 months) of animal behavior in the field. These echinoids are abundant in coastal environments of the northwestern Pacific; however, their ranges overlap only partially, in the southern part of the Sea of Japan washing the coasts of Korea, Japan and the Primorye region of Russia, where the lower and upper geographical boundaries of *S. intermedius* and *M. nudus* occur, respectively (see for review Agatsuma, 2013a, 2013b; Kafanov & Pavlyuchkov, 2001). In the Sea of Japan, these sea urchins are common species in benthic communities inhabiting hard substrates at a depth of up to 25 m (Kafanov & Pavlyuchkov, 2001). Morphologically, *S. intermedius* and *M. nudus* differ from each other in respect to the color (brownish-black in *M. nudus* and brownish-grey in *S. intermedius*) and spine length and hardness (hard sharp spines of up to 30 mm in *M. nudus* and less hard spines of up to 8 mm in *S. intermedius*). We hypothesized that these sea urchin species, presumably sharing an evolutionary history but differing morphologically, might display different behavioral responses to various disturbing factors such as high hydrodynamic activity and predation.

## Materials and Methods

### Study areas, sea urchins and video recording experimental setup

Field experiments were conducted in the northwestern Sea of Japan: Kievka Bay (42.830°N, 133.691°E) and Alekseev Bay (42.981°N, 131.730°E). Kievka Bay, with a width of 8.3 km and a length of 3.3 km, is typical of the southeastern coast of the Primorye region of Russia. Being open to prevailing summer winds from the southeast to the southwest, the bay is characterized by high wave activity. The studies here were performed at a depth of 6 m on relatively flat bedrock surrounded by large stones. Alekseev Bay has a width of 0.8 km and a length of 1.3 km and wave activity here is high only under winds from the north, a phenomenon that is quite rare in summer. The studies here were performed at a depth of 2 m on a flat bottom covered with medium sized gravel. Within both bays, water depth is weakly influenced by tidal activity: an irregular semidiurnal tide has a maximum amplitude of approximately 50 cm (Zhadan, Vaschenko & Ryazanov, 2019).

In both bays, the density of *S. intermedius* and *M. nudus* in the areas adjacent to the experimental installations was 1 to 2 ind. m^−2^ (Zhadan, Vaschenko & Ryazanov, 2019). In addition, to balance the density of the species in the study areas, approximately 300 specimens of *S. intermedius* were collected in the bays in a radius of 100–200 m from the experimental installations and placed at a distance of 10–20 m from them. The video observation of sea urchins’ behaviors was performed with TLC200 Pro (Brinno Incorporated, Taipei City, Taiwan) time-lapse video cameras mounted on steel stanchions approximately 1 m above the bottom. Time-lapse videos were taken in 1 min intervals at a resolution of 1280 × 720 pixels. The cameras were installed in such a way that the size of the field of view was approximately 1.5 × 1.0 m. During the night, the cameras’ fields of view were illuminated by LED lamps (1 W) which were synchronized with the cameras by flash LED indicator. The illumination duration was 1 s.

To attract sea urchins in the field of view of video cameras, flat mesh containers filled with the kelp *Saccharina japonica* (Laminariales, Phaeophyta; hereafter simply laminaria) were used. It is known that laminaria stimulates foraging activity of *M. nudus* and *S. intermedius* (Machiguchi, 1987; Machiguchi, Mizutori & Sanbonsuga, 1994). The containers, each composed of two steel frames and mesh stretched on them, 1.1 × 0.75 × 0.01 m in size, were filled with laminaria and placed in the cameras’ field of view. Each container (hereafter feeder) contained approximately 30 kg of the kelp. Sea urchins of both species themselves found the feeders and populated them within 2–3 days. All sea urchins that were in the cameras’ field of view on all the feeders were taken into account for further video analysis. The steel frames of the containers were pressed down by stones with a diameter of 20–40 cm to protect against wave activity.

The indicator of the onset of sea urchins’ alarm response was the escape or redistribution of sea urchins in the field of view of the cameras, and the indicator of the end of the alarm reaction was the return of sea urchins to their original spatial distribution pattern. Such a design of the experiments allowed us to clarify (1) the features of sea urchins’ behavior in the presence of abundant palatable food and under different weather conditions, (2) the frequency of predator attacks under natural conditions and (3) the temporal patterns of the complete behavioral response of sea urchins to a natural predator attack and simulated predator attack (crushed conspecifics or heterospecifics) in sea urchin species with different morphological characteristics.

### Video recording analysis

The recorded videos were viewed frame by frame. We counted the numbers of specimens of each sea urchin species in the each camera field of view both in the absence and in the presence of disturbing factors. To access the grouping behavior of the sea urchins, we calculated the mean group size as the ratio of the total number of individuals in the cameras’ field of view to the number of associations (Hagen & Mann, 1994). Following Vadas et al. (1986), we distinguished between sea urchin associations and aggregations. Each group of sea urchins in two-dimensional groupings, including individuals suspected of being in tactile contact (there was no visible space between them) and single individuals, was considered a separate association.

To determine the mean group size for both sea urchin species, the video frames were randomly selected over the periods of calm weather. Only the video frames showing not more than 30 specimens (43 frames for each species) were chosen for the mean group size calculation in order to avoid crowding effects when sea urchins might be in tactile contact due to their high density on the feeders. Under such a limitation, no groups of three or more sea urchins in cohesive three-dimensional groupings (aggregations, according to Vadas et al., 1986) were observed in our study.

Sea urchins’ movement was analyzed using the free software, “Tracker”, for video analysis (http://www.opensourcephysics.org/items/detail.cfm?ID=7365). The cell size (2 × 2 cm) of the feeder mesh was used as a scale. We tracked and measured sea urchin displacement with an interval of 1 min. Following Lauzon-Guay, Scheibling & Barbeau (2006), we defined a step as the distance between two successive positions of the sea urchin (1 min apart), a stop as an interval when sea urchin remains stationary during at least 1 min (two successive frames) and a move as the distance between two successive stops which can be composed of one or more steps. The mean locomotion speed was calculated as total distance passed divided by total time.

### Long-term video recording of sea urchins’ behavior

**Figure 1:**
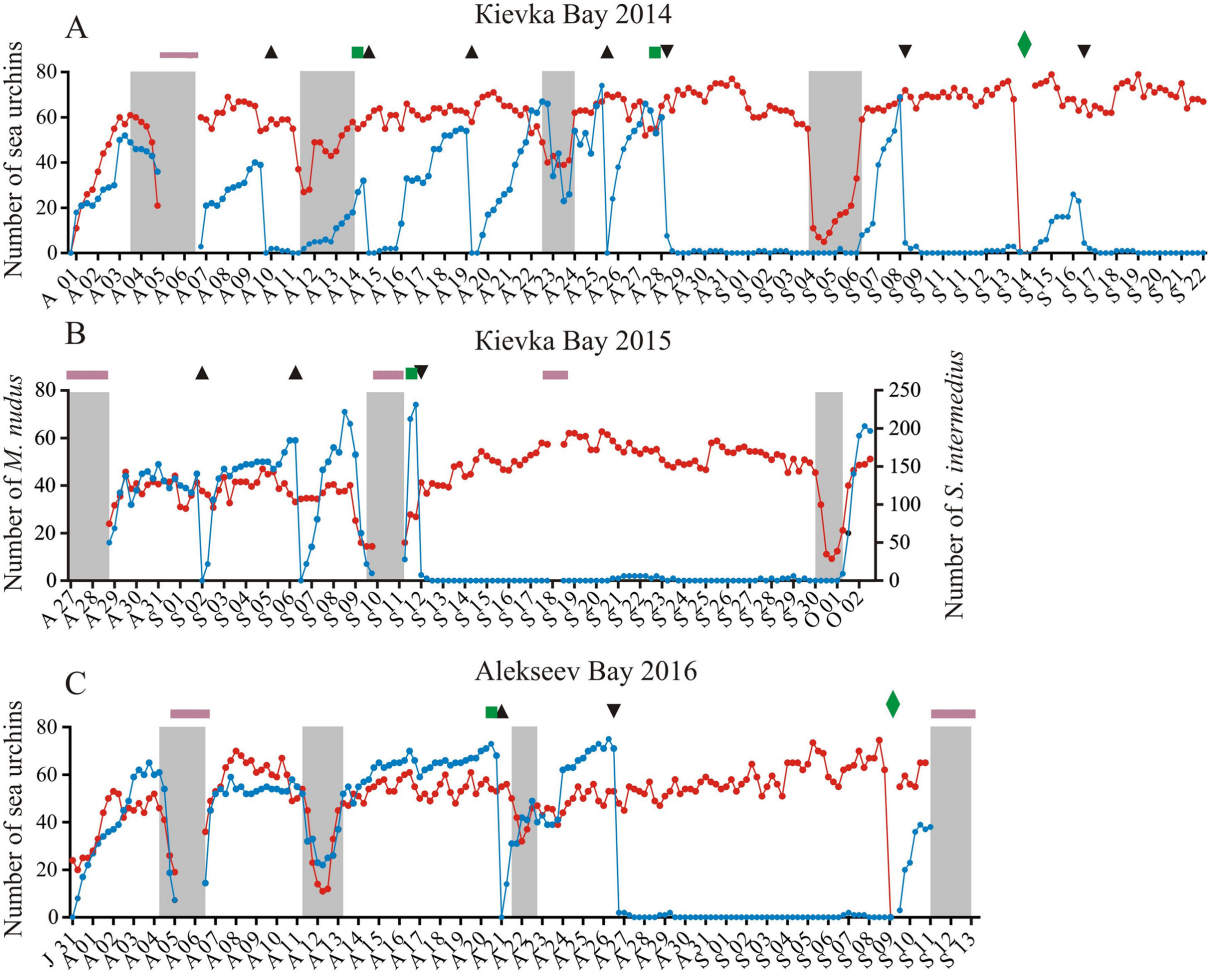
Temporal dynamics of the numbers of sea urchins *Mesocentrotus nudus* and *Strongylocentrotus intermedius* in long-term experiments. The experiments were conducted in: (A) Kievka Bay, 2014; (B) Kievka Bay, 2015; (C) Alekseev Bay, 2016. Blue and red circles connected by lines denote the numbers of *M. nudus* and *S. intermedius*, respectively, presented as the sum of all sea urchins of the given species on four feeders per each 6 h of observations (see Table S1 for original data). Triangles indicate the time points when sea urchins *M. nudus* were removed from the feeders. Upside down triangles denote the time points when sea urchins *M. nudus* were crushed near the feeders. Green squares denote the time points when the feeders were changed. Green rhombuses indicate the time points when the feeders were changed after the mimicking of stormy weather conditions. Shaded areas denote storm periods. Solid violet horizontal lines indicate periods of poor visibility because of high water turbidity. *X*-axis: month and date.

We conducted three separate video recordings in Kievka Bay (during 51 days in August–September 2014 and 37 days in August–October 2015) and in Alekseev Bay (during 46 days in July–September 2016). In these experiments, four cameras were used and four feeders were arranged in pairs in such a way that the direction of the tidal currents coincided with the direction of the long sides of the feeders. The distances between the long sides of the feeders were 0.4 m and the distance between the pairs of the feeders was 2 m. The feeders were replaced every 15–20 days (Fig. 1) when approximately 80% of laminaria was consumed by sea urchins. Sea urchins were carefully transferred from the old feeders to the feeders with fresh laminaria. It took approximately 5 min to change one feeder.

We tested for behavioral differences between *M. nudus* and *S. intermedius* in presence of a large quantity of palatable food but under different weather conditions and different types of predation threat: (1) under calm weather conditions, (2) under stormy weather conditions, (3) under predation risk associated with the presence of several sea star species and (4) under predation risk associated with simulated predator attack (crushed individuals of *M. nudus*). The numbers of specimens of each sea urchin species were counted per each 6 h of observations throughout all three long-term experiments, both in the absence and in the presence of disturbing factors (see Table S1 for original data).

The stormy periods were determined by several signs which are clearly visible on video recordings: (1) the oscillation of the feeder surface and (2) the increase in suspended particles movement and water turbidity. In addition, an increase in wave height during storm was recorded by the depth sensor of a multi-parameter RBRXRX-620 datalogger (Sea and Land Technologies Pte, Singapore) which measured the sea level every 10 min (Fig. S1).

We compared movement parameters (the number and length of the steps and moves, the entire displacement, the number and duration of the stops, the entire stationary period and mean speed) of both sea urchin species under calm and stormy weather conditions. For this purpose, the distances traversed by randomly selected 10 sea urchins of each species during 240 min before the storm and in the beginning of the storm (Fig. S1) were tracked and measured with an interval of 1 min (see Tables S2 and S3 for raw data). Further tracking showed that a half of observable sea urchins left the cameras’ field of view during approximately 10 h after the beginning of measurements. Locomotion speed during escape was calculated as total distance passed during the period from the beginning of active movement of the sea urchin to its disappearance from the field of view divided by the total time of escape (see Tables S2 and S3 for raw data).

Predation risk was associated with several species of sea stars that were present simultaneously with sea urchins on the feeders or with simulation of predation attack (crushed individuals of *M. nudus*). To determine behavioral responses of *S. intermedius* to starfish attacks on the conspecifics which took place on September 5 and 21, 2015 (Table S1), we measured the distances of 13 sea urchins closest to the site of the attack (i.e., their entire displacement) during approximately 100 h after the beginning of the attack with the intervals from 2 h to 4–16 h. For these periods, the total numbers of *S. intermedius* specimens as well as the individuals of two starfish species, *P. pectinifera* and *L. fusca*, which were in contact with the prey, were counted.

Our pilot experiments showed that *M. nudus* rapidly left the area of the bottom where conspecifics were crushed and avoided it for a long time. There was no similar reaction to crushed *S. intermedius* specimens. Furthermore, *S. intermedius* exhibited no visible responses both to crushed conspecifics and heterospecifics. Therefore, we focused on the study of the phenomenon of *M. nudus* response to crushed conspecifics. We compared how quickly *M. nudus* populated the feeders in the presence and in the absence of crushed conspecifics. In the first case, 10 *M. nudus* specimens with test diameters of 50–60 mm were crushed in the middle between the pairs of the feeders when *M. nudus* were present on them (five experiments in total; we did not include the data of one experiment into statistical analysis because it might be influenced by the experiment on storm imitation conducted 3 days earlier). In the second case, all *M. nudus* specimens were removed from the cameras’ fields of view and transferred to a distance of approximately 10 m from the feeders (seven experiments in total; we did not include the data of three experiments into statistical analysis because they might be influenced by the previous or next storm). In both cases, we estimated the temporal dynamics of the numbers of sea urchins (with time intervals of 1 h and 6 h in the first and second cases, respectively). To test our assumption that the storm contributes to the return of sea urchins *M. nudus* to the feeders, two experiments mimicking stormy conditions were conducted several days (6 and 12 in 2014 and 2016, respectively, Table S1) after *M. nudus* left the feeders in response to crushed conspecifics. The feeders were removed, and several scuba divers actively swam in the area of the experimental installation. After that, the feeders with fresh laminaria were placed in their original place. These procedures took 30–40 min. The average sea urchin numbers for 1 day before crushing and 2 days after storm imitation were determined.

### Short-term video recording of sea urchins’ behavior

A set of experiments, each with a duration of approximately 6 days, was conducted in Alekseev Bay from July to September 2016. The experiments were designed to elucidate behavioral patterns of *M. nudus* and *S. intermedius* in response to an alarm stimulus (crushed conspecifics or heterospecifics). In each of these experiments, two video cameras and two feeders were used. Two days after the feeder placement, when 30–40 sea urchins of both species were gathered on the feeder surface, five specimens of *S. intermedius* or *M. nudus* were crushed at a distance of 20 cm from the feeder’s short side. Three to four days after simulation of the predation threat, all the sea urchins were removed from the cameras’ field of view, and the experiment was repeated on the other feeder with fresh laminaria and with sea urchins not previously used. In total, four experiments of this kind were carried out with each of sea urchin species used as simulated prey. During 22 h before and 55 h after the treatment, we estimated the numbers of sea urchins as well as the temporal dynamics of the mean group size (with time intervals from 1 to 4 h) with one exception: we failed to assess the temporal dynamics of the mean group size of sea urchins *M. nudus* in response to crushed conspecifics due to fast escape of *M. nudus*.

To determine movement patterns of sea urchins in response to crushed con- and heterospecifics, we measured step length and locomotion speed before and after treatment. Additionally, we measured with intervals of 6–9 min the distances of 10 sea urchins closest to the site where simulated attack was performed. The exception was a case with response of *M. nudus* to crushed conspecifics because sea urchins rapidly left the cameras’ field of view. Duration of sea urchin tracking was from 200 to 1200 min due to the species specific response of sea urchins to the alarm signal.

### Statistical analysis

To analyze the species-specific and treatment-specific differences in *M. nudus* and *S. intermedius* behavior, the data sets on the numbers of sea urchins, mean group sizes as well as sea urchins’ movement parameters were formed and tested for normal distribution (D’Agostino and Pearson omnibus normality test, *P* < 0.05). Normally distributed data were further analyzed by parametric tests (unpaired *t*-test, 1-way ANOVA). In a case of abnormally distributed data, non-parametric tests were used (Mann–Whitney test, Kruskal–Wallis test followed by Dunn’s multiple comparisons). To analyze the temporal dynamics of the numbers of both sea urchins in the experiments on the response to crushed conspecifics or heterospecifics as well as in the experiments on the repopulation the feeders by sea urchins *M. nudus* after their removal, linear or nonlinear regression (curve fit) were used. All statistical analyses were run using GraphPad Prism v. 6.0. The details regarding raw data and statistics are presented in the Supplementary Materials. In all the Figures, the data relating to *M. nudus* is in blue color and the data relating to *S. intermedius* is in red color.

### Procedural controls

In our in situ experiments we did not use the procedural controls which are usually applied in the experiments with animals contained in tanks or cages, such as “food presence–food absence”, “animal treatment/manipulation–imitation of animal treatment/manipulation”. First, both sea urchin species exhibited clear food search behavior, and the absence of food strongly stimulated them to migrate to the place where the food is present. Second, we minimized manipulations in our experiments that could affect the results and conclusions. A negligible displacement of water masses near a feeder associated with sea urchins’ crushing as well as the swimming of the diver, who served the installation, above the feeder did not cause escape reactions of both sea urchin species similar to those during storm. The experiments mimicking stormy conditions were conducted in the absence of the feeders. Both in long-term and short-term experiments, we used short-term periods (from 1 to 3 days) just before the treatments as proper procedural controls, and compared quantitative parameters (sea urchins’ number and mean group size) obtained for these periods with those obtained for the periods after the treatments (storms, starfish attacks or crushed sea urchins).

## Results

### General characteristics of sea urchins’ behavior

During the recording periods in 2014, 2015 and 2016 (134 days in total), 24 ± 25 (mean ± SD) of *M. nudus* specimens and 78 ± 42 of *S. intermedius* specimens were in the field of view of the video cameras (Figs. 1A–1C; Table S1). During the periods without any treatment (storms, *M. nudus* specimens removal or crushing, starfish attacks), the numbers of *M. nudus* and *S. intermedius* were higher, 50 ± 15 and 85 ± 42, respectively. The size composition of the two echinoid species was slightly different. Only large adults of *M. nudus* with a test diameter of 62.2 ± 7.5 mm (mean ± SD) were present in the cameras’ field of view, whereas among the adult *S. intermedius* with test diameters from 37 to 74 mm (64.4 ± 4.3 mm), there was a small number (up to 12%) of juveniles with test diameters of 10–15 mm. On the surface of the feeders, both sea urchin species were relatively evenly distributed in one plane and did not form aggregates (three-dimensional groups); however, they formed associations (dense two-dimensional groups). Grouping behavior in *S. intermedius* was expressed to a greater extent than in *M. nudus*: when from 14 to 25 of individuals were present on the surface of the feeders, the mean group size of *S. intermedius* was approximately 2 times higher than that of *M. nudus* (2.27 ± 0.4 vs. 1.12 ± 0.1, Mann–Whitney test, *U* = 0, *P* < 0.0001; see Table S4 for raw data and statistics).

Both sea urchin species displayed so called “covering behavior” but it was more pronounced in *S. intermedius* than in *M. nudus*. The debris covering sea urchin aboral surfaces consisted mainly of pieces of the algae such as *Ulva fenestrata* Ruprecht, 1840 and *Desmarestia viridis* (O.F. Müller) J.V. Lamouroux, 1813 in June–July, and seagrass *Zostera marina* Lamouroux in the end of September. During these periods, from 87% to 100% of *S. intermedius* individuals were decorated compared to from 0% to 18% for *M. nudus*.

### Behavioral responses of sea urchins to increased wave activity

**Figure 2:**
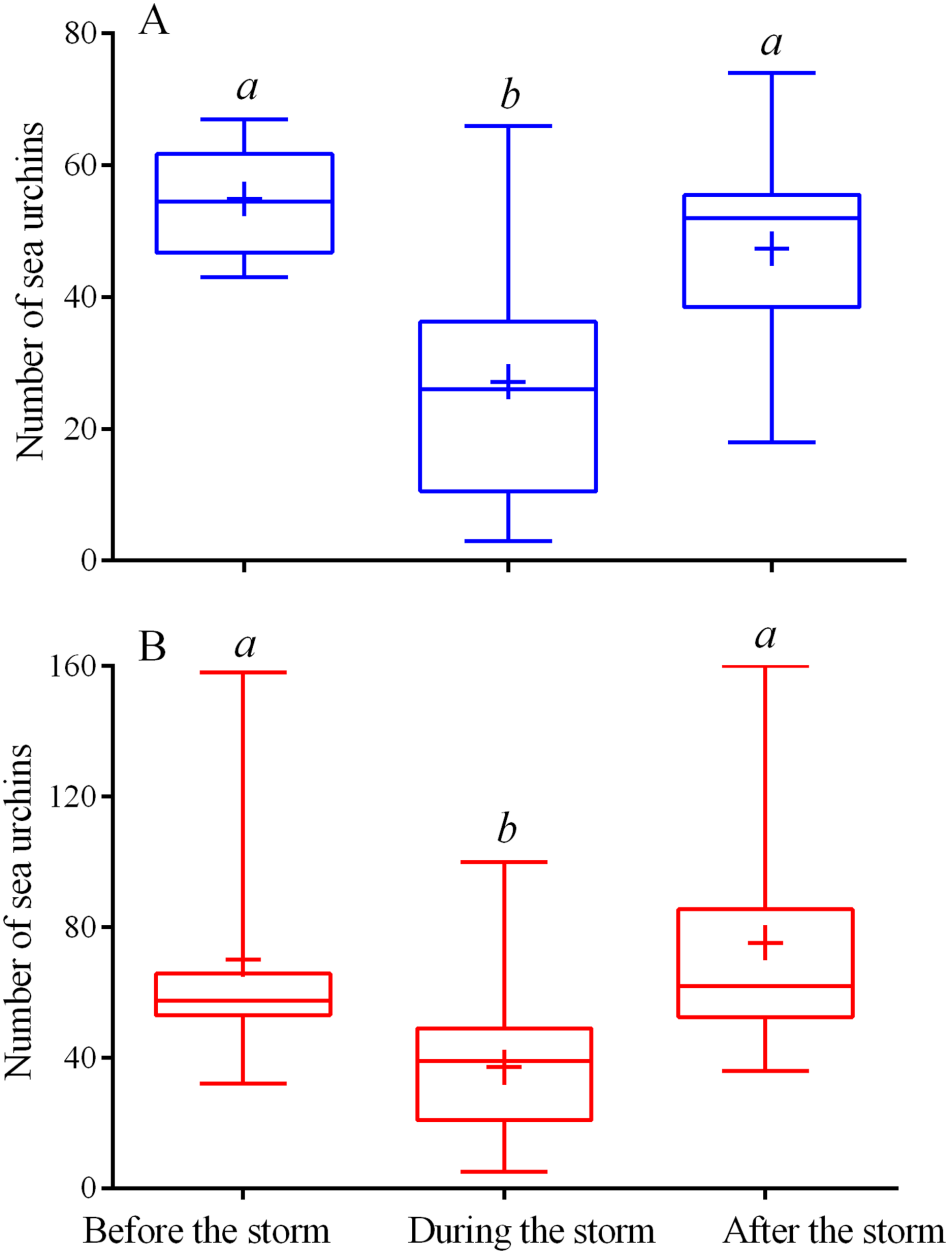
Changes in the numbers of sea urchins *Mesocentrotus nudus* (A) and *Strongylocentrotus intermedius* (B) in response to the stormy weather. Range (whiskers), upper and lower quartile (box), mean (+), and median (solid line) of the numbers of sea urchins before, during and after the storm periods are presented. Different lowercase letters above the boxes indicate significant differences in sea urchin numbers: (A) the differences between “before the storm” and “during the storm” numbers are significant at *P* < 0.0001, the differences between “during the storm” and “after the storm” numbers are significant at *P* < 0.001 (1-way ANOVA followed by Tukey’s multiple comparisons test), (B) the differences between “before the storm” and “during the storm” numbers, “during the storm” and “after the storm” numbers are significant at *P* < 0.0001 (Kruskal–Wallis test followed by Dunn’s multiple comparisons test). See Tables S5 and S6 for raw data and statistics.

During the storms, the number of sea urchins of both species in the cameras’ field of view sharply decreased (Figs. 1A–1C; see Table S1 for raw data). On the eve of the storm periods, there were 54 ± 9 (mean ± SD) of *M. nudus* specimens and 76 ± 37 of *S. intermedius* specimens whereas during the storms, the average numbers for both species (26 ± 15 and 34 ± 21 for *M. nudus* and *S. intermedius*, respectively) were significantly lower (Fig. 2; see Tables S5 and S6 for raw data and statistics). Approximately 1 day after the storm, sea urchins of both species restored their numbers on the feeders (Figs. 1A–1C and 2; Tables S5 and S6).

Both sea urchin species exhibited similar patterns of movement under conditions of calm weather. The average numbers of moves and stops were not significantly different between species, however, the moves of *S. intermedius* were shorter and consisted of higher number of shorter steps whereas the locomotion speed and entire distance traveled were significantly higher in *M. nudus* (Tables 1 and 2; see also Table S7 for interspecies comparison).

**Table 1:** Parameters of movement activity of the sea urchin *Mesocentrotus nudus* under calm and stormy weather. Data are presented as Mean ± SEM (n = 10) and the range (in the parentheses) for 240 min interval.

Parameter	Calm weather	Stormy weather	Statistics
Number of steps	110 ± 12 (50–160)	21 ± 3 (9–35)	*t*_10_ = 7,314; df = 9.883; *p* < 0.0001
Step length, cm	0.71 ± 0.08 (0.10–9.54)	0.29 ± 0.02 (0.10–1.34)	*t*_10_ = 4.966; df = 10.26; *p* = 0.0005
Number of moves	28 ± 2 (22–35)	13 ± 2 (5–23)	*t*_10_ = 5.915; df = 18; *p* < 0.0001
Move length, cm	2.99 ± 0.58 (0.10–56.5)	0.57 ± 0.16 (0.10–8.37)	Mann–Whitney *U* = 5.0; *p* = 0.0002
Entire distance traversed, cm	76.90 ± 11.86 (34.91–146.40)	5.87 ±0.7 (3.91–9.97)	*t*_10_ = 5.98; df = 9.063; *p* = 0.0002
Number of stops	28 ± 2 (22–35)	14 ± 2 (5–24)	*t*_10_ = 5.773; df = 17.41; *p* < 0.0001
Stop duration, min	4.82 ± 0.54 (1–43)	19.69 ± 3.02 (1–87)	*t*_10_ = 4.845; df = 9.566; *p* = 0.0008
Entire stop duration, min	130.0 ± 11.87 (80–190)	217.9 ± 2.73 (205–231)	*t*_10_ = 7.219; df = 9.95; *p* < 0.0001
Speed, cm min^−1^	0.32 ± 0.05 (0.15–0.61)	0.03 ± 0.003 (0.02–0.04)	*t*_10_ = 6.054; df = 9.056; *p* = 0.0002

**Table 2:** Parameters of movement activity of the sea urchin *Strongylocentrotus intermedius* under calm and stormy weather. Data are presented as Mean ± SEM (*n* = 10) and the range (in the parentheses) for 240 min interval.

Parameter	Calm weather	Stormy weather	Statistics
Number of steps	160 ± 10 (110–211)	57 ± 12 (18–141)	*t*_10_ = 6.606; df = 17.49; *p* < 0.0001
Step length, cm	0.28 ± 0.03 (0.10–5.46)	0.33 ± 0.08 (0.10–4.14)	Mann–Whitney *U* = 33.5; *p* = 0.2233
Number of moves	31 ± 2 (16–39)	26 ± 3 (13–39)	*t*_10_ = 1.147; df = 18; *p* = 0.2662
Move length, cm	1.65 ± 0.35 (0.10–39.72)	0.65 ± 0.17 (0.10–37.7)	Mann–Whitney *U* = 13.0; *p* = 0.0038
Entire distance traversed, cm	44.28 ± 5.56 (29.73–88.85)	16.10 ± 4.3 (3.80–45.76)	Mann–Whitney *U* = 12.0; *p* = 0.0029
Number of stops	31 ± 2 (18–39)	26 ± 3 (12–38)	*t*_10_ = 1.331; df = 15.56; *p* = 0.2023
Stop duration, min	2.54 ± 0.26 (1–29)	8.63 ± 1.70 (1–80)	*t*_10_ = 3.402; df = 9.431; *p* = 0.0058
Entire stop duration, min	80.1 ± 10.1 (29–130)	183.2 ± 11.97 (99–222)	*t*_10_ = 6.592; df = 17.49; *p* < 0.0001
Speed, cm min^−1^	0.19 ± 0.02 (0.12–0.37)	0.07 ± 0.02 (0.02–0.19)	Mann–Whitney *U* = 11.0; *p* = 0.0019

Both sea urchin species responded to increased wave activity by a sharp decrease in the number of steps, length of one move and entire distance traveled (Tables 1 and 2; see also Table S7 for interspecies comparison). At the same time, the duration of one stop increased approximately 3 and 4 times in *S. intermedius* and *M. nudus*, respectively, and the average proportions of time sea urchins spent stationary were 76% and 91%, respectively, against 33% and 54% under calm weather. The average locomotion speeds during storm conditions were as low as 0.07 and 0.03 cm min^−1^ in *S. intermedius* and *M. nudus*, respectively. During escape, sea urchin speeds sharply increased and averaged 0.82 ± 0.19 cm min^−1^ (step range of 0.1−7.02 cm) in *S. intermedius* and 1.76 ± 0.30 cm min^−1^ (step range of 0.1−11.81 cm) in *M. nudus*, these were, respectively, 4.3 and 5.5 times higher than those under calm weather (see Tables S2 and S3 for raw data).

### Behavioral response of *S. intermedius* to starfish attack

Over three periods of our studies, only three cases of the attacks of the sea stars (*Patiria pectinifera* (Muller & Troschel, 1842) and *Lethasterias fusca* Djakonov, 1931) on single individuals of *S. intermedius* were recorded (5, 10 and 21 September, 2015, Table S1) whereas no attacks of predators on sea urchins *M. nudus* were observed. Judging by the presence of injuries and abnormal behavior (low motor activity and body position with the oral surface upward), only sick or damaged individuals of *S. intermedius* have been attacked by sea stars (Fig. S2A). It is possible that the appearance of damaged *S. intermedius* specimens that have lost a significant part of the spines was due to typhoon “Goni”, which occurred on August 27–29, 2015 in the northwestern Sea of Japan.

**Figure 3:**
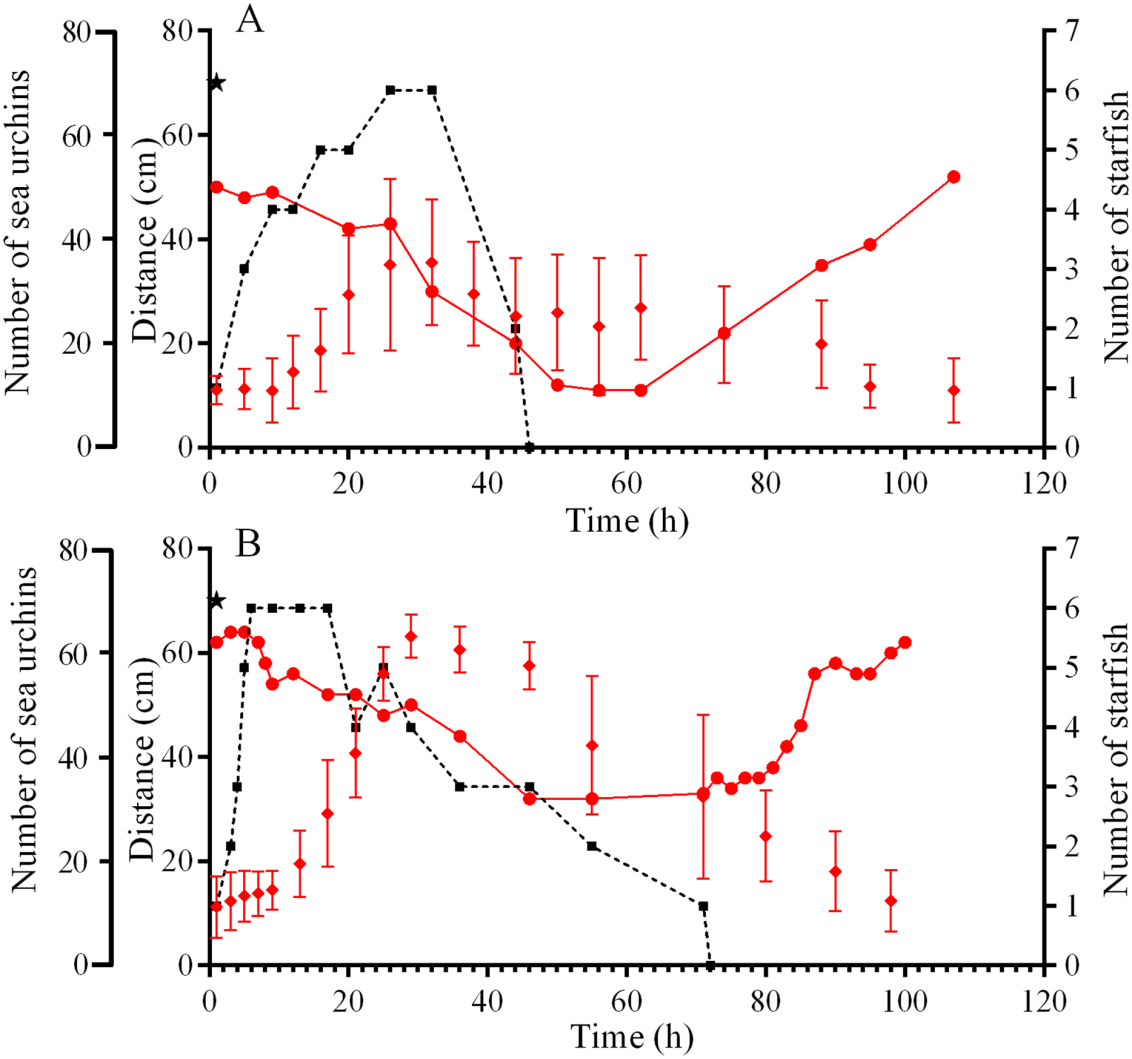
Movement activity of the sea urchins *Strongylocentrotus intermedius* in response to starfish attacks. (A) Starfish attack on September 5, 2015. (B) Starfish attack ****on September 21, 2015. ****Red rhombuses denote sea urchin distances from the site of the attack, mean ± SD (*n* = 13). Black squares connected by dashed line denote the sum number of two species of sea stars (*Patiria pectinifera* and *Lethasterias fusca*) at the site of the attack. Red circles connected by solid line denote the number of sea urchins in the cameras’ field of view. Time of the beginning of the attack is indicated by a black asterisk.

On September 10, sea urchin reaction to a predator attack could not be traced due to low visibility caused by the storm. The behaviors of sea urchins *S. intermedius* during starfish attacks on September 5 and 21, 2015 were slightly different. On September 5, one *S. intermedius* specimen was consumed by 1–5 individuals of *P. pectinifera* and one individual of *L. fusca* in the center of one of the four feeders for 45 h (Fig. 3A; Fig. S2). Within 2 h after the beginning of the attack, most of the sea urchins left the surface of the feeder and formed several groups on the tops and at the base of nearby stones (Fig. S2B). The number of sea urchins in the cameras’ field of view was relatively stable during the first 12 h, and sea urchin distances from the site of attack did not change much (Fig. 3A). Then, the distances began to increase sharply, and their maximum coincided with maximum number of sea stars consuming an ailing specimen (Fig. 3A). A total of 26 h after the beginning of starfish attack, the number of sea urchins in the cameras’ field of view began to decrease, and after 50 h, there remained approximately 30% of sea urchins (Fig. 3A; Fig. S2C). The number of sea urchins on the feeder began to increase between 16 and 28 h after the starfish left the sea urchin remains, and this coincided with gradual decrease of sea urchin distances from the site of attack (Fig. 3A). In general, the alarm reaction of *S. intermedius* from the onset of the starfish attack to restoration of the initial sea urchin population on the surface of the feeder (Fig. 3A; Fig. S2D) lasted for approximately 90 h.

On September 21, a starfish attack occurred at the short edge of the feeder. Sea urchin distances from the site of attack were almost unchanged during the first 9 h and then sharply increased, and this coincided with maximum number of sea stars (*P. pectinifera* and *L. fusca*) consuming an ailing specimen (Fig. 3B). After 24 h, no *S. intermedius* specimens remained closer than 40 cm from the site of attack. They formed associations on the feeder and the nearest stones. Eight hours after the beginning the attack, the number of sea urchins in the cameras’ field of view began to decrease, and after 55 h, there remained approximately 50% of sea urchins. Consumption of the prey by the sea stars lasted 70 h. Restoration of sea urchin abundance and distribution on the feeder began 10 h after the sea stars left the remains of the prey. The total duration of the sea urchin alarm reaction was 88 h (Fig. 3B).

It should be noted that the sea stars *P. pectinifera* and *L. fusca* were constantly present on the feeders. The starfish *Asterias amurensis* Lutken, 1871, *Distolasterias nipon* Döderlein, 1902, *Lysastrosoma anthosticta* Fisher, 1992 and *Aphelasterias japonica* Bell, 1881 also often appeared on the feeders. With a few exceptions, these starfish did not cause visible reactions in healthy sea urchins. The behavior of *P. pectinifera* was the most aggressive. In one case, during 28 min, *P. pectinifera* attacked an *S. intermedius* specimen, which lost approximately 20% of its spines, but finally, it was left alone. In two cases, sea stars *P. pectinifera* completely crawled on *S. intermedius* individuals in such a way that starfish mouth was located directly above the sea urchin’s anal orifice. After 11 and 15 min in the first and second cases, respectively, the sea stars left the potential prey, which indicates that a healthy sea urchin can effectively resist the penetration of a starfish stomach through the anus.

### Behavioral responses of *M. nudus* and *S. intermedius* to crushed *M. nudus* specimens

**Figure 4:**
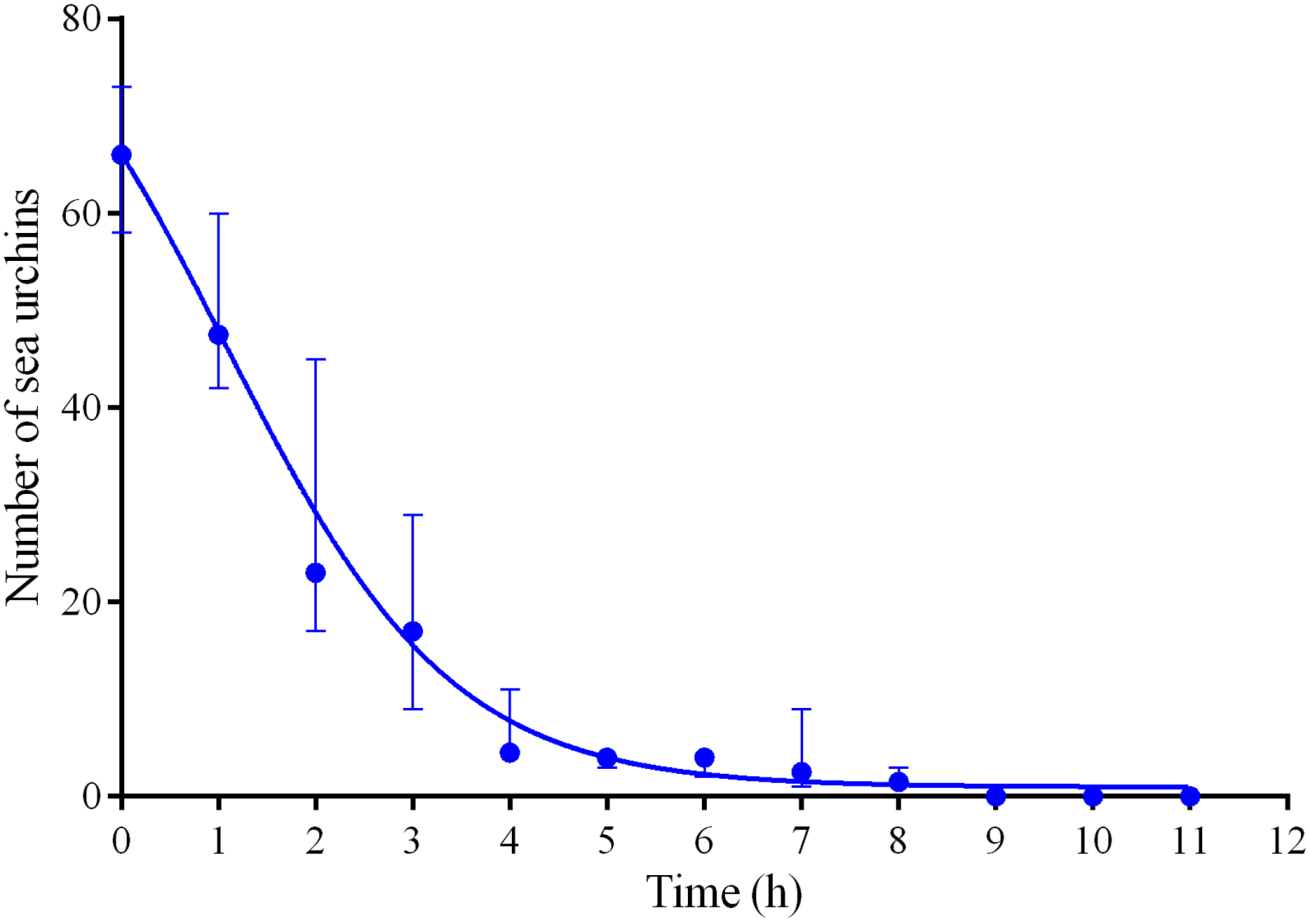
Changes in the numbers of sea urchins *Mesocentrotus nudus* during escape in response to crushed conspecifics in long-term experiments of 2014–2016. The data of four experiments conducted during long-term recordings of 2014–2016 (Figs. 1A–1C; Tables S1 and S8) are presented as median and range of the number of *M. nudus* per 1 h after crushing of conspecifics. Nonlinear regression is significant (*R*^2^ = 0.9454, see Table S8 for raw data and statistics).

**Figure 5:**
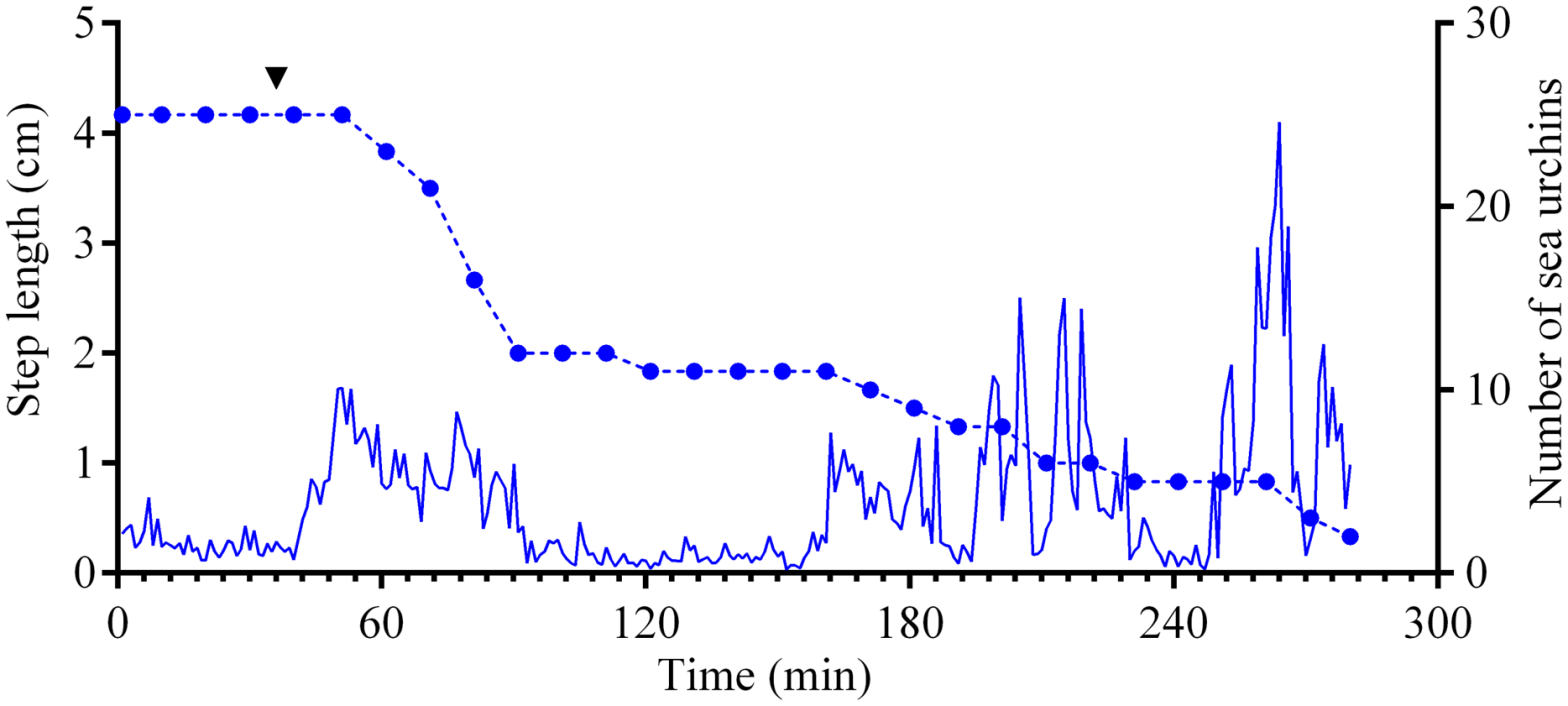
Movement activity of the sea urchins *Mesocentrotus nudus* in response to crushed conspecifics. Blue solid line denotes the average step length of sea urchins (*n* = 10). Blue circles connected by dashed line denote the number of sea urchins in the cameras’ field of view. Time point of treatment is denoted by upside down triangle.

Four experiments conducted during long-term recordings of 2014–2016 (Figs. 1A–1C; Table S1) showed that after the conspecifics were crushed near the feeders, approximately 90% of *M. nudus* individuals left the cameras’ field of view during 4 h (Fig. 4; see also Table S8 for raw data and statistics). During this period, a sharp increase in the average step length just after the treatment was recorded (Fig. 5). There were two time intervals with the highest locomotion speed, the first 55 min after the treatment when a half of *M. nudus* specimens left the cameras’ field of view, and the last 128 min when the rest of sea urchins escaped (Fig. S3A). These intervals were interrupted by the relatively stable 1 h period when sea urchins almost stopped moving (Fig. 5).

Nine hours after the beginning of the experiment, there were no *M. nudus* specimens on the feeders (Fig. 4; Table S8) and then, during a much longer period (7–19 days), only single *M. nudus* individuals appeared (Figs. 1A–1C). The restoration of the initial sea urchin numbers on the feeders occurred only after the next storm event.

The imitation of stormy weather also contributed to the return of *M. nudus* after sea urchins left the feeders in response to presentation of crushed conspecifics. Sea urchins were absent on the feeders during approximately 5 and 13 days in 2014 and 2016, respectively, and came back within 2 days after intensive swimming and replacement of the feeders; however, their number was lower than that before the experiments (Figs. 1A–1C; Fig. S4; Table S1).

**Figure 6:**
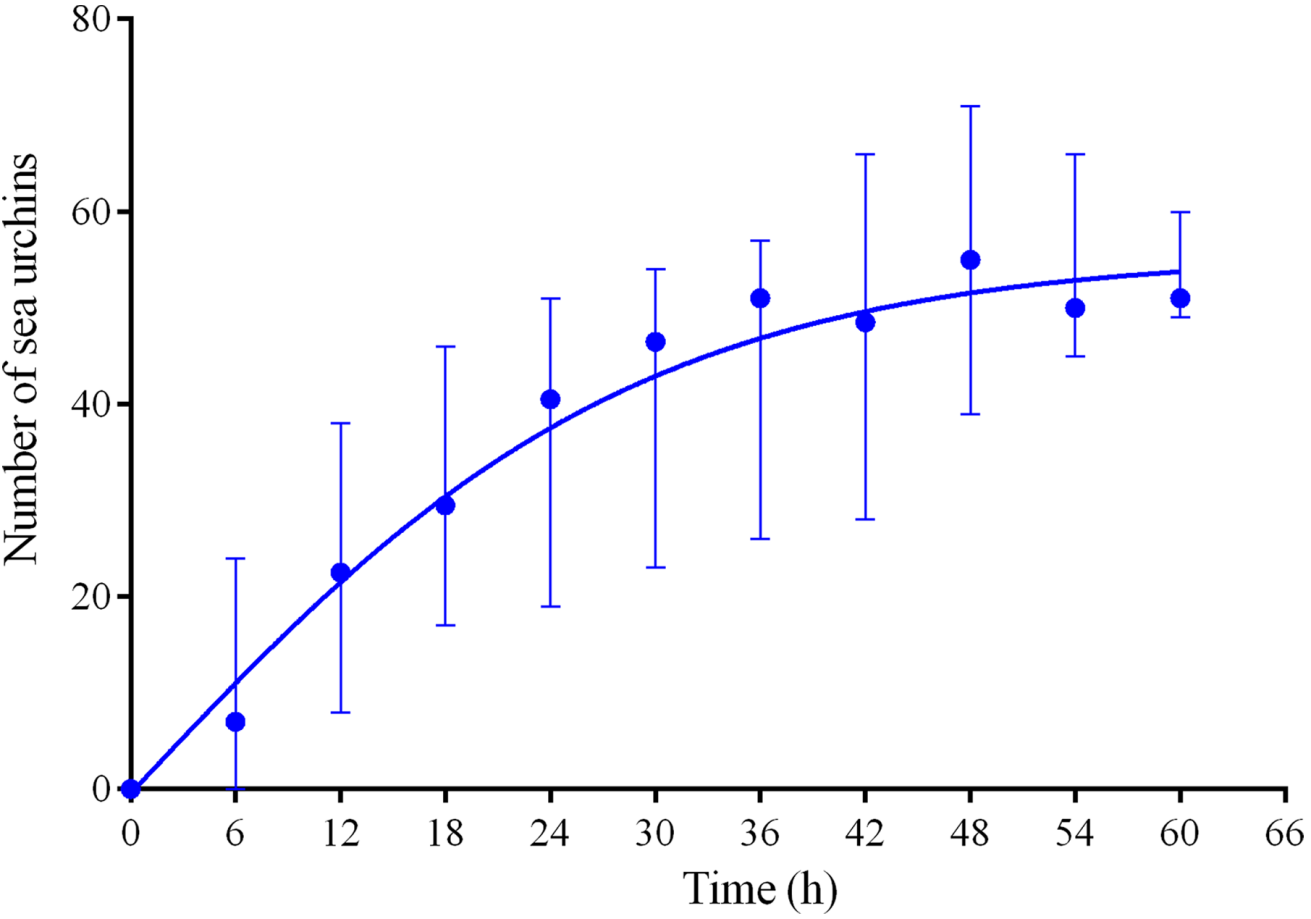
Temporal dynamics of repopulation of the feeders by sea urchins *Mesocentrotus nudus* after their removal in long-term experiments of 2014–2016. The data of four experiments conducted during long-term recordings of 2014–2016 (Figs. 1A–1C; Tables S1 and S9) are presented as median and range of the number of *M. nudus* per 6 h. Nonlinear regression is significant (*R*^2^ = 0.731, see Table S9 for statistics).

Four experiments conducted during long-term recordings of 2014–2016 (Figs. 1A–1C; Table S1) demonstrated that, in the absence of crushed conspecifics, sea urchins *M. nudus* came back 2–3 days after their removal from the surfaces of the feeders (Fig. 6; see Table S9 for raw data and statistics).

Sea urchins *S. intermedius* did not leave the cameras’ field of view during the experiments with presentation of crushed *M. nudus* specimens (Figs. 1A–1C; Table S1); moreover, statistical analysis revealed small but significant increase in *S. intermedius* numbers in three cases when *M. nudus* was absent on the feeders (see Table S10 for statistics). Spatial pattern of *S. intermedius* remained almost unchanged with the exception of one case: after the *M. nudus* specimens were crushed, sea urchins *S. intermedius* avoided the surface of the feeder for 24 h and were among the stones opposite of the crushed *M. nudus*.

### Behavioral responses of *M. nudus* and *S. intermedius* to crushed conspecifics and heterospecifics

**Figure 7:**
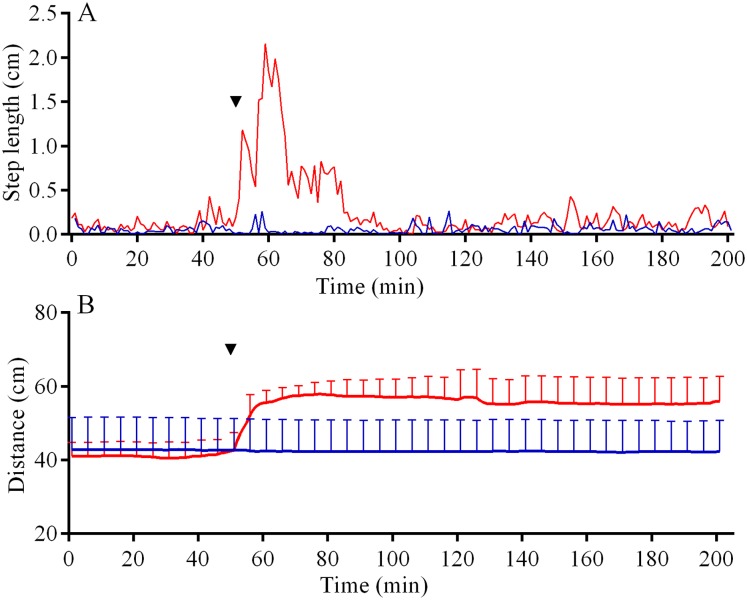
Movement activity of the sea urchins *Mesocentrotus nudus* (blue lines) and *Strongylocentrotus intermedius* (red lines) in response to crushed heterospecifics. (A) The changes in the average step length of sea urchins (*n* = 10). (B) The changes in sea urchin distances from the site of simulated attack, mean ± SD (*n* = 10). SD is shown for every sixth measurement. Time of the treatment is denoted by upside down triangle.

In the 6 days short-term experiments, *M. nudus* exhibited a strong avoidance reaction in response to crushed conspecifics similar to that in long-term observations (Fig. S5A; see Table S11 for raw data and statistics). When *S. intermedius* individuals were crushed near the feeders, behavior of sea urchins *M. nudus* remained unchanged, as evidenced by the absence of significant changes in such indicators as the number of sea urchins in the cameras’ field of view (Fig. S5B; see Table S12 for raw data and statistics) and mean group size (Fig. S5C; see Table S13 for raw data and statistics). Analysis of *M. nudus* movement activity also revealed no differences in the average step length (Fig. 7A), distance from the site of simulated attack (Fig. 7B) and locomotion speed (Fig. S3C).

**Figure 8:**
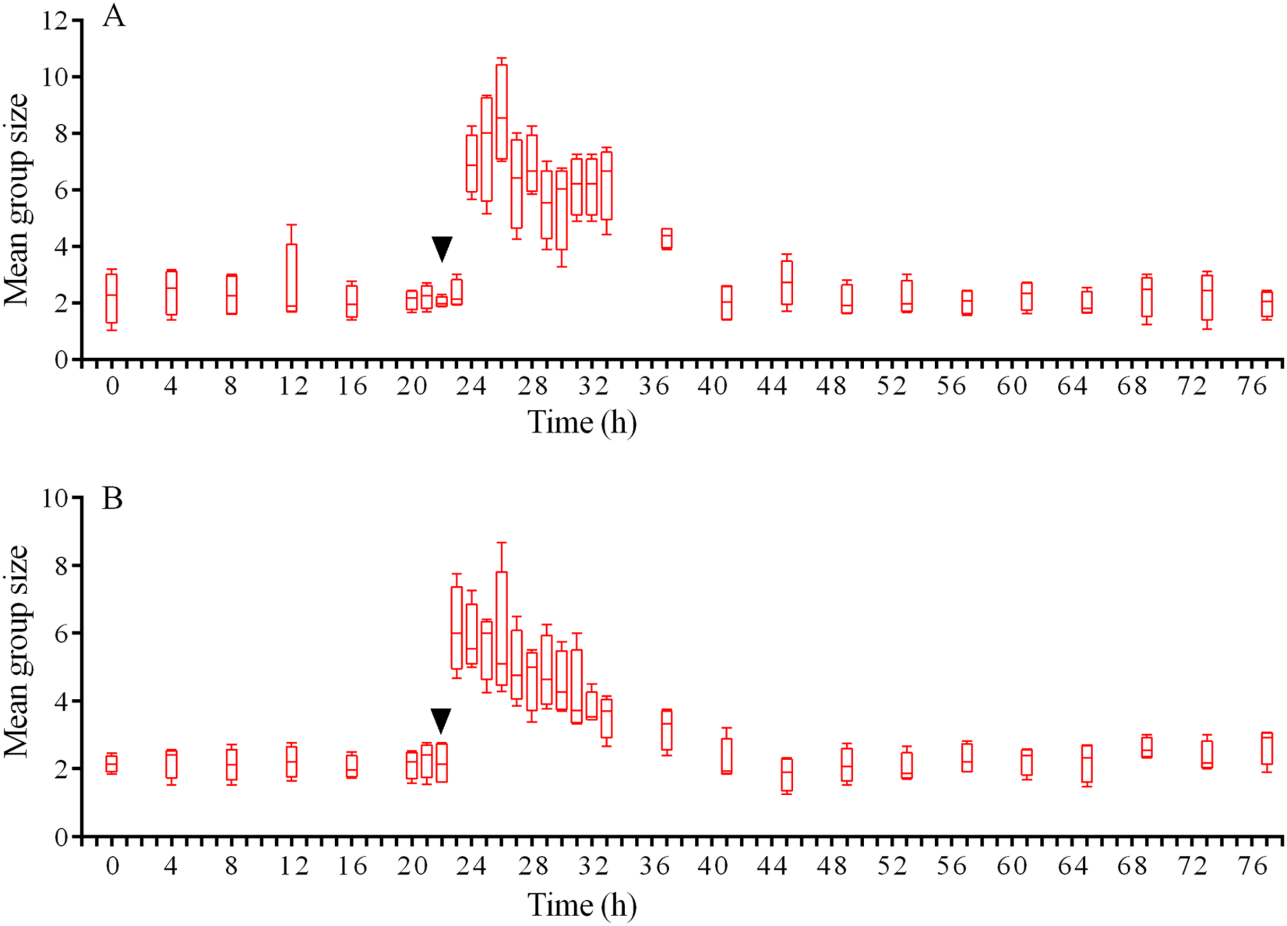
Temporal dynamics of the mean group size of sea urchins *Strongylocentrotus intermedius* in response to simulated predator attack. (A) Response of *S. intermedius* to crushed conspecifics. (B) Response of *S. intermedius* to crushed specimens of the sea urchin *Mesocentrotus nudus*. The mean group size is presented as box-whisker plot showing the median (solid line), range (whiskers) and upper and lower quartiles (box). Upside down triangles denote the time points when sea urchins were crushed near the feeders. See Tables S14 and S17 for raw data and statistics.

**Figure 9:**
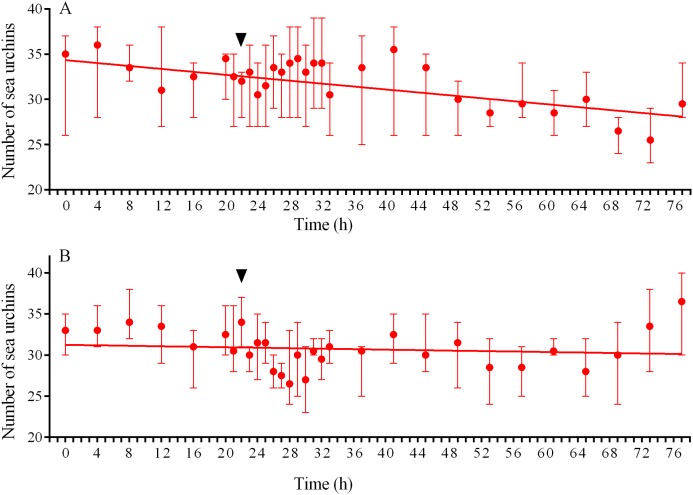
Temporal dynamics of the numbers of sea urchins *Strongylocentrotus intermedius* in response to simulated predator attack. (A) Response of *S. intermedius* to crushed conspecifics. Linear regression is significant (*P* < 0.0001, see Table S15 for raw data and statistics). (B) Response of *S. intermedius* to crushed specimens of the sea urchin *Mesocentrotus nudus*. Linear regression is not significant (*P* = 0.3485, see Table S18 for raw data and statistics). The data are presented as median and range. Time of the treatment is indicated by upside down triangle.

In response to crushed conspecifics, *S. intermedius* moved towards the opposite side of the feeder and formed associations there. Before the experiment, the mean group size was 2.25 ± 0.75 (mean ± SD for 21 h of observation), and this parameter was 2.5-fold higher (5.85 ± 2.18) during the 14 h period after the stimulus was presented (Fig. 8A; see Table S14 for raw data and statistics). The changes in the number of sea urchins in the cameras’ field of view were not very obvious (Fig. 9A); however, linear regression showed significant decrease in this parameter (*P* < 0.0001; see Table S15 for raw data and statistics). The associations were positioned in such a way that sea urchins were partly on the feeder and partly outside it. On average, approximately 40% of the sea urchins, both grouped and alone, were located in the immediate vicinity outside the feeders. Judging by the restoration of the original spatial distribution of sea urchins on the feeder (the initial mean group size), the duration of the alarm reaction of *S. intermedius* was approximately 18 h (Fig. 8A).

**Figure 10:**
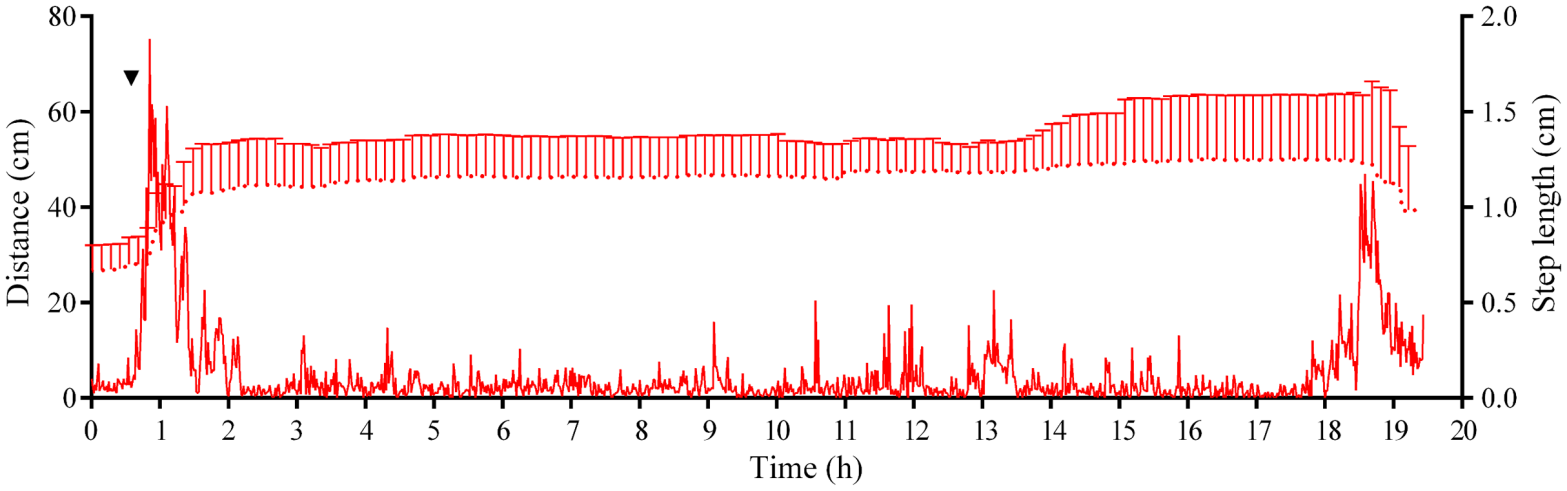
Movement activity of the sea urchins *Strongylocentrotus intermedius* in response to crushed conspecifics. Red dotted line denotes sea urchin distance from the site of simulated attack, mean ± SD (*n* = 10). SD is shown for every ninth measurement. Red solid line denotes the average step length of sea urchins (*n* = 10). Time of the treatment is indicated by upside down triangle.

Analysis of *S. intermedius* movement revealed two time intervals of the highest activity, each approximately 1.5 h in duration: the first when sea urchins formed associations and the second when these associations dispersed (Fig. 10; Fig. S3B; see Table S16 for raw data). Between these two peaks of activity, there was prolonged period of approximately 16 h, when the average step length and locomotion speed were relatively low, and the distance from the site of simulated attack was almost unchanged.

Judging by the dynamics of the mean group size, response of *S. intermedius* to crushed individuals of *M. nudus* was generally similar to the response to crushed conspecifics (Fig. 8B; see Table S17 for raw data and statistics). Movement analysis showed that just after the treatment, there was an increase in the average step length (Fig. 7A), distance from the site of simulated attack (Fig. 7B) and locomotion speed (Fig. S3D). A vast majority of sea urchins did not leave the cameras’ field of view (Fig. 9B) and linear regression showed no significant decrease in this parameter (*P* = 0.3485; see Table S18 for raw data and statistics). The original spatial distribution of sea urchins on the feeder was restored within approximately 20 h after the beginning of simulated attack (Fig. 8B).

## Discussion

The present work is the first study analyzing long-term, around-the-clock behavior of sea urchins exposed under field conditions to both calm and stormy weather and to presentation of two competing stimuli, food and predation threat, which are assumed to be key factors influencing species survival. Due to the natural turbidity of sea water, video camera cannot register the initial stages of the sea urchin alarm reaction, namely, extension of the tube feet and movement of spines, which are manifested in the first seconds or minutes after sea urchin exposure to an alarm signal (Morishita & Barreto, 2011; Urriago, Himmelman & Gaymer, 2011). At the same time, the method of continuous time-lapse video recording allowed analysis of the long-term dynamics of the spatial distributions of sea urchins under natural conditions when both stimuli were presented. Previously, several authors applied video recording in the field to quantify sea urchins at the kelp grazing front (Lauzon-Guay & Scheibling, 2007) and to determine sea urchin movement patterns (Dumont, Himmelman & Robinson, 2007; Lauzon-Guay, Scheibling & Barbeau, 2006); however, they used separate time-lapse video sequences, each lasting several hours. We showed that two sea urchin species, different in morphology and living under similar conditions, exhibited distinctly different strategies for avoiding predation in terms of response duration and behavioral patterns.

### Sea urchin response to hydrodynamics

In the absence of predation threat, *S. intermedius* and *M. nudus* also showed somewhat different behavioral patterns. Under calm weather, *S. intermedius* much more often than *M. nudus* exhibited the covering behavior and tended to group on the food substrate. Despite general patterns of movement (intermittent locomotion, characterized by moves interspersed with pauses) in both species, *M. nudus* spent more time stationary but moved at approximately 2 times higher speed than *S. intermedius*, resulting in the higher distance traversed. However, both species are highly mobile, and under conditions of calm sea and presence of food, *M. nudus* and *S. intermedius* would be able to move on average 4.6 and 2.7 m per day, respectively (calculated based on the average locomotion speeds). These average distances are similar to those observed for the sea urchins *Strongylocentrotus droebachiensis* (0.4–1.72 m; Dumont, Himmelman & Russell, 2006), *Toxopneustes roseus* (1.65 and 2.49 m; James, 2000), *Tripneustes ventricosus* (3.7 and 8.8 m; Tertschnig, 1989) and *Diadema antillarum* (3.7 m; Tuya, Martin & Luque, 2004).

The results of both laboratory and field experiments evidence that sea urchins are capable to sense a change in hydrodynamic activity and react by changing the behavior. In our study, both sea urchins responded to the stormy weather, firstly, by decreasing the movement activity up to almost complete stop and secondly, by leaving the food. It is noteworthy that during escape under the stormy conditions, sea urchins can move on average 4–5-fold faster than during feeding under calm weather.

It is known that with increasing water velocity (higher than approximately 15 cm s^-1^), sea urchins decrease their displacement and cease feeding both under laboratory (Kawamata, 1998; Frey & Gagnon, 2016; Cohen-Rengifo et al., 2018; Tamaki, Muraoka & Inoue, 2018) and field (Lissner, 1980; Dance, 1987; Siddon & Witman, 2003; Dumont, Himmelman & Russell, 2006; Dumont, Himmelman & Robinson, 2007) conditions. Escape behavior was also observed in laboratory flume experiments: at flow velocity ≤30 cm s^−1^, sea urchins moved in a downstream direction whereas at 35–45 cm s^−1^, individuals moved in an upstream direction (Morse & Hunt, 2013; Cohen-Rengifo et al., 2018). However, escape response of sea urchins to wave-induced benthic water flow in situ has been poorly documented. Dance (1987) observed that during a period of turbulence lasting several hours, movement of *P. lividus* was significantly oriented to the deep water with lower hydrodynamic activity. Lauzon-Guay & Scheibling (2007) found that *S. droebachiensis* density at the grazing front decreased when wave action increased and suggested that the “whiplash effect” of the swaying kelp prevented sea urchins from climbing onto kelp plants. In our study, the kelp was packed into mesh containers and formed a kind of soft substrate. We believe that sea urchins *M. nudus* and *S. intermedius*, being able to sense an oscillation of the substratum and/or increasing water flow, reduce the movement activity and then make a decision to stay close to the food or escape. We noted that in the case of the weaker storm, only a part of sea urchins left the food source. Remaining *S. intermedius* individuals gathered into groups at the base of the feeders whereas *M. nudus* specimens were on the surface of the feeders. However, unfortunately, we did not measure a velocity of water flow and, therefore, cannot correlate it with sea urchin movement activity. Further studies are thus required to elucidate such a correlation.

### Sea urchin response to predation threat

During this study, no successful starfish attacks on healthy sea urchins were observed for either species, and only three cases of consuming of single ailing *S. intermedius* individuals by several starfish specimens (*P. pectinifera* and *L. fusca*) were recorded. Taking into account the laboratory experimental data that the starfish, *L. anthosticta* and *P. pectinifera* in particular, are predators of *M. nudus* (see for review Agatsuma, 2013b), we further analyzed the video records of 2017 captured by six video cameras in the course of another project. During 44 days, 132 ± 46 of *M. nudus* individuals were in the cameras’ field of view and no cases of predator attacks were recorded (Zhadan, Vaschenko & Ryazanov, 2019). Considering also that both sea urchin species showed only a weak response to sea stars even during direct contact, we can conclude that none of six starfish species observed on the feeders (*P. pectinifera*, *L. fusca*, *A. amurensis*, *D. nipon*, *L. anthosticta* and *A. japonica*) are specialized predators of *S. intermedius* and *M. nudus*. Most likely, starfish perform the function of scavengers.

The alarm response of *S. intermedius* to predation depended on the type of alarm signal and involved several phases. The sea urchins: (1) moved away from a source of threat (so called “flight response”), (2) exhibited grouping behavior forming dense two-dimensional groups close to the food source, (3) left the food source and (4) restored the initial spatial distribution on the food source. Phase three was most pronounced when the alarm signals were the attack of starfish on ailing specimens or simulated attack with crushed conspecifics: approximately 50 h after the beginning of starfish attack, there remained only 30–50% of sea urchins on the feeders. When the alarm signal was a simulated attack with crushed heterospecifics, phase three was much weakly pronounced or absent.

Sea urchins *M. nudus* exhibited a fast (during 4 h) escape (flight response) and prolonged (up to 19 days) avoidance of the source of attractive food near which the conspecifics were crushed. Such a long fear effect of the alarm signal associated with crushed conspecifics is probably due to the marking of the area with substances released by injured sea urchins. At the same time, *M. nudus* exhibited no responses to damaged heterospecifics.

Escape of sea urchins from an alarm source is the most well-documented first phase of the alarm response of sea urchins (Snyder & Snyder, 1970; Parker & Shulman, 1986; Scheibling & Hamm, 1991; Campbell et al., 2001; Hagen, Andersen & Stabell, 2002; Vadas & Elner, 2003; Urriago, Himmelman & Gaymer, 2011; Wirtz & Duarte, 2012). It has been shown that the alarm reaction in sea urchins started within a few minutes of exposure to waterborne chemosensory cues from some potential predators (fish, lobsters, crabs, sea stars, gastropods), as well as from crushed prey, conspecifics or heterospecifics. Generally, escape response was most pronounced in the experiments with damaged conspecifics.

To date, responses to chemosensory cues from damaged conspecifics have been demonstrated in several sea urchin species: *Diadema antillarum* (Kintzing & Butler, 2014a; Snyder & Snyder, 1970), *S. droebachiensis* (Hagen, Andersen & Stabell, 2002; Mann, 1982), *Echinometra viridis* and *Lytechinus williamsi* (Parker & Shulman, 1986), *Echinometra lucunter* (Morishita & Barreto, 2011; Parker & Shulman, 1986), *Echinus esculentus* and *Psammechinus miliaris* (Campbell et al., 2001), *Lytechinus variegatus* and *Tripneustes ventricosus* (Vadas & Elner, 2003), and *Arbacia lixula* and *Sphaerechinus granularis* (Wirtz & Duarte, 2012). It should be noted, however, that Parker & Shulman (1986) did not find an alarm reaction to extracts of conspecifics when analyzing sea urchin motion for 1 min in *Eucidaris tribuloides*, *T. ventricosus* and *L. variegatus* living in long, dense seagrass that provided protection from detection by predators, and *D. antillarum* occupying crevices.

In our studies, both *M. nudus* and *S. intermedius* exhibited a phased escape response to crushed conspecifics: (1) a sharp increase in locomotion speed just after presentation of an alarm signal lasting for 1–1.5 h and resulting in an increase of a distance from a threat source and grouping close to a food source (*S. intermedius*) or escape of approximately half of specimens (*M. nudus*), (2) deceleration lasting for approximately 1 h in *M. nudus* and 16 h in *S. intermedius* and (3) the second increase in locomotion speed lasting for approximately 1.5 h and resulting in association dispersion and repopulation of feeders (*S. intermedius*) or escape of all remaining specimens (*M. nudus*). Previously, Vadas & Elner (2003) found in the field experiments that two sympatric tropical sea urchin species, *L. variegatus* and *T. ventricosus*, demonstrated an initial burst of speed followed by a gradual deceleration up to relatively stable level in response to conspecific alarm cues. However, these movement reactions were much shorter and lasted only 2 min each.

The most striking difference of these sea urchins in the escape responses to conspecific alarm cues was that *M. nudus* exhibited fast and total escape whereas only a part of *S. intermedius* individuals left a food source and the remaining specimens formed associations on the feeder and in close vicinity to it. Our results showed that grouping behavior is a common phase of the alarm response in *S. intermedius* but not in *M. nudus*. As it was shown in laboratory and field studies, a number of sea urchin species are capable of forming dense groups on a food substrate (Bernstein, Schroeter & Mann, 1983; Bernstein, Williams & Mann, 1981; Garnick, 1978; Pearse, 2006; Vadas & Elner, 2003). Bernstein, Williams & Mann (1981), Bernstein, Schroeter & Mann (1983) found that the presence of predators (lobsters *Homarus americanus* or crabs *Cancer irroratus*) in laboratory aquariums or in field cages triggered the formation of *S. droebachiensis* aggregations that were larger than groups of feeding and non-feeding sea urchins in the absence of predators. The researchers interpreted such aggregation behavior as a defense mechanism of *S. droebachiensis* against predation. Vadas et al. (1986), however, did not find a tendency to form aggregations in the same species in the presence of predators (decapods *H. americanus*, *C. irroratus* and sea star *Asterias vulgaris*) and suggested that grouping of sea urchins in tank corners or on tank walls/cage mesh may be an experimental artifact caused by the accumulation of sea urchins near artificial obstacles that prevented them from escaping a predator. This point of view was supported by other studies on interactions between sea urchins *S. droebachiensis* and their predators in field and laboratory experiments (Harding & Scheibling, 2015; Scheibling & Hamm, 1991). In addition, Vadas & Elner (2003), investigating the reactions of sympatric sea urchins *L. variegatus* and *T. ventricosus* to simulated predator attacks in field experiments, also found no formation of sea urchin groups in response to an alarm signal and concluded that the flight response is the primary, and perhaps only defensive behavior employed by these species. However, our results showed that two other sympatric sea urchin species (*M. nudus* and *S. intermedius*) exhibit distinctly different behavioral response strategies to predation risk: *M. nudus* employs fast escape and prolonged avoidance of dangerous area while *S. intermedius* employs both grouping and escape behaviors.

The duration of the alarm response of *S. intermedius* (from the appearance of the alarm signal to the return to feeding) was different under different conditions. It was the longest (approximately 90 h) after the attack of sea stars on ailing individuals. The eating of prey lasted from 45 h to 70 h, and after that, from 20 h to 45 h passed before sea urchins restored their original arrangement on the feeder. Considering that semidiurnal tidal cycles and constant wave activity took place in the study areas, there is little reason to believe that a waterborne chemical cue from the primary source (injured prey) could have persisted. The secondary source of the alarm signal could be the products of predator metabolism (Scherer & Smee, 2016). For example, black sea urchin *E. lucunter* is able to distinguish sea stars feeding on conspecifics or closely related species (Morishita & Barreto, 2011). For predatory fish, it has been shown that substances that cause the alarm response in the prey can remain active after passing through the digestive tract (Manassa & McCormick, 2012). In addition, it is likely that waterborne chemical cues from predators and/or injured prey may be sorbed on the bottom sediments and gradually released, thereby increasing the time of the alarm reaction in prey.

Based on the above data, it may be assumed that the duration of the alarm reaction of sea urchins depends on two main factors: (1) the duration of the release of substances from predators and/or injured prey to the environment and (2) the time during which sea urchins can detect these substances sorbed on the sediment. In addition, species-specific previous learning might also be a factor determining different behavioral patterns in sea urchin species (Ferrari, Wisenden & Chivers, 2010).

In our study, sea urchins *M. nudus* exhibited unique prolonged avoidance behavior in response to crushed conspecifics and this behavior has not been described before in sea urchins and other echinoderms. The ability of *M. nudus* to avoid a site of predation for up to 19 days and return to the food source only after a storm indicates that crushed *M. nudus* specimens released some stable substances that marked the bottom for a long time and served as an alarm signal and that the disruption and removal of the upper sediment layer during the storm probably contributed to the removal of the alarm signal. The experiments with storm imitation support this suggestion.

### Possible mechanisms underlying the difference in sea urchin alarm responses

The mechanisms underlying different patterns of the alarm responses in cohabiting sea urchin species are not yet understood. We believe that both the ability of *S. intermedius* to form associations close to a food source and the ability of *M. nudus* to leave the area of predation risk for a long period are useful evolutionary adaptations that enhance the likelihood of species survival under permanent pressure from visual predators.

We suggest that sea urchin *S. intermedius* uses camouflage to protect itself from visual predators because a group of these sea urchins forms a gray spot of irregular shape decorated with algae, which is more difficult to be identified from air or under water than a single object with a regular round shape. Furthermore, a solitary sea urchin is easier to be captured by a diving predator. At the same time, such camouflage cannot be effective for sea urchin *M. nudus* because its black color is in high contrast to the color of the bottom, whereas leaving the area occupied by a predator increases the chances of *M. nudus* survival.

It is well known that in temperate waters, the most active consumers of sea urchins that are able to control their abundance are the sea otter *Enhydra lutris* (Duggins, 1980; Estes & Duggins, 1995; Watson & Estes, 2011) and a number of bird species, mainly gulls (Guillemette, Ydenberg & Himmelman, 1992; Himmelman & Steele, 1971; Hori & Noda, 2007; Merkel et al., 2007; Wootton, 1995). Wootton (1995) compared the densities of sea urchin *Strongylocentrotus purpuratus* in several places in a lower intertidal zone both exposed to bird predators (glaucous-winged gulls *Larus glaucescens*, American black oyster catchers *Haematopus bachmani* and northwestern crows *Corvus caurinus*) and protected from birds by cages and showed that sea urchin abundance was 59% lower after 1 year and 45% lower after 2 years in the presence of bird predators compared to the absence of bird predators. For *S. intermedius*, the most abundant avian predators are carrion crow *Corvus corone* and a few gull species that are able to consume a large number of sea urchins, more than 4,000 specimens per 1 h (Hori & Noda, 2007).

Data on the geographical distributions of *S. intermedius* and *M. nudus* (Agatsuma, 2013a, 2013b; Bazhin, 1998; Kafanov & Pavlyuchkov, 2001) and the sea otter (Kenyon, 1969) give evidence that the ranges of these species may have partially overlapped in the past, but at present, the overlapping of this predator–prey habitat seems more likely for temperate-boreal species, *S. intermedius*, which inhabits the Asian Pacific coastal waters from the Kamchatka Peninsula southward to the Korean Peninsula and from the Russian coast eastward to the Japanese Islands. The sea urchin *M. nudus* is a subtropical species and coastal waters of the Sea of Japan near Russia (Primorye Region) and Japan (northern Hokkaido) represent the northern part of its range, whereas for the sea otter, the northern Hokkaido represented the southern boundary of its range in the northwestern Pacific until the 18th century, before fur hunting began (Wilson et al., 1991).

Sea otters and predatory birds prefer sea urchins of medium and large size, that is, adult specimens contributing to population reproduction (Estes & Duggins, 1995; Guillemette, Ydenberg & Himmelman, 1992; Himmelman & Steele, 1971; Hori & Noda, 2007). The defensive behaviors of sea urchins *S. intermedius* and *M. nudus* could have formed mainly under the pressure of these predators. Due to natural selection, the individuals that could avoid predation attacks survived, and useful genetic traits have been passed from generation to generation in the form of different defensive behaviors.

## Conclusion

Our results show that cohabiting sea urchin species, *S. intermedius* and *M. nudus*, which were monitored in their natural environment under conditions of food abundance display both similar and different behavioral responses to hydrodynamics and predation threat. The most interesting findings are the following: (1) under calm weather, *S. intermedius* but not *M. nudus* tended to group on the food substrate; movement patterns of both sea urchins were similar but *M. nudus* exhibited the higher locomotion speed and distance traveled; (2) both sea urchins responded to increased wave activity by a sharp decrease in the movement activity up to almost complete stop and then made a decision to stay close to the food or escape; (3) several sea star species failed to predate on healthy sea urchins of both species and only a few starfish attacks on ailing *S. intermedius* specimens were successful; (4) the alarm response of *S. intermedius* depended on the type of alarm signal (consumption of ailing conspecifics by starfish or simulated attack) and included the formation of dense groups close to the food source; (5) the alarm response of *S. intermedius* lasted approximately 90 h and 20 h for starfish attacks on ailing conspecifics and for simulated attacks (crushed conspecifics or heterospecifics), respectively; (6) *M. nudus* responded to crushed conspecifics only and exhibited no grouping behavior but displayed fast escape (during 4 h) and prolonged (up to 19 days) avoidance of the food source; (7) both sea urchins exhibited a phased escape response to crushed conspecifics consisting of a sharp increase in locomotion speed just after presentation of the alarm signal followed by deceleration and the second increase in locomotion speed associated with repopulation of feeders (*S. intermedius*) or complete escape (*M. nudus*); (8) damaged specimens of *M. nudus* released some stable alarm substances. Furthermore, our results show the benefits of using continuous time-lapse video recording to study the long-term behavioral responses of sea urchins to different disturbing factors such as high hydrodynamic activity and predation threat. Considering the important ecological role of sea urchins as grazers of marine plants, data on the duration of the fear response in sea urchin species, that is, the periods when their foraging activity is inhibited, may be of greatest use in mathematical modeling of the marine ecosystem.

## Supplemental Information

10.7717/peerj.8087/supp-1 Supplemental Information 1 The numbers of sea urchins *Mesocentrotus nudus* and *Strongylocentrotus intermedius* in long-term experiments conducted in Kievka Bay in 2014 and 2015 and in Alekseev Bay in 2016. ‘nd’ indicates the periods of poor visibility because of high water turbidity.

10.7717/peerj.8087/supp-2 Supplemental Information 2 The distances (cm) traveled by sea urchins *Mezocentrotus nudus* during 240 min under conditions of calm (A) and stormy (B) weather and during escape under conditions of stormy weather (С). (A) The distances (cm) traveled by sea urchins *M. nudus* during 240 min under conditions of calm weather. (B) The distances (cm) traveled by sea urchins *M. nudus* during 240 min under conditions of stormy weather. (C) The distances (cm) traveled by sea urchins *M. nudus* during escape under conditions of stormy weather.

10.7717/peerj.8087/supp-3 Supplemental Information 3 The distances (cm) traveled by sea urchins *Strongylocentrotus intermedius* during 240 min under conditions of calm (A) and stormy (B) weather and during escape under conditions of stormy weather (C). (A) The distances (cm) traveled by sea urchins *S. intermedius* during 240 min under conditions of calm weather. (B) The distances (cm) traveled by sea urchins *S. intermedius* during 240 min under conditions of stormy weather. (C) The distances (cm) traveled by sea urchins *S. intermedius* during escape under conditions of stormy weather.

10.7717/peerj.8087/supp-4 Supplemental Information 4 Mean group size for *Mesocentrotus nudus* and *Strongylocentrotus intermedius* in the absence of any treatment in long-term experiments of 2014–2016.

10.7717/peerj.8087/supp-5 Supplemental Information 5 Changes in the numbers of sea urchins *Mesocentrotus nudus* in response to the stormy weather in long-term experiments of 2014–2016. The data are extracted from Table S1. The stormy periods when *M. nudus* specimens were absent on the feeders due to their removal or escape response to presentation of crushed conspecifics were excluded from statistical analysis.

10.7717/peerj.8087/supp-6 Supplemental Information 6 Changes in the numbers of sea urchins *Strongylocentrotus intermedius* in response to the stormy weather in long-term experiments of 2014–2016. The data are extracted from Table S1. The stormy periods which directly followed the experiments with application of other disturbing stimuli such as removal of *M. nudus* from the feeders or presentation of crushed *M. nudus* specimens were excluded from statistical analysis.

10.7717/peerj.8087/supp-7 Supplemental Information 7 Comparison of the parameters of movement activity in the sea urchins *Mesocentrotus nudus* and *Strongylocentrotus intermedius* under calm and stormy weather. Data are presented as mean ± SEM (*n* = 10) and the range (in the parentheses) for 240 min interval.

10.7717/peerj.8087/supp-8 Supplemental Information 8 Escape response of sea urchins *Mesocentrotus nudus* to crushed conspecifics in long-term experiments of 2014–2016. The data of four experiments conducted during long-term recordings of 2014–2016 (Table S1) are presented as the numbers and percentages of sea urchins per 1 h for the period of 11 h after the crushing of conspecifics near the feeders. The average number of sea urchins for 24 h period before the beginning of the experiment is considered to be 100%. The data are extracted from Table S1. Experiment conducted on September 16, 2014 was not included into statistical analysis because it might be influenced by the experiment on storm imitation conducted 3 days earlier, on September 13, 2014.

10.7717/peerj.8087/supp-9 Supplemental Information 9 Temporal dynamics of repopulation of the feeders by sea urchins *Mesocentrotus nudus* after their removal in long-term experiments of 2014–2016. The data from four experiments conducted during long-term recordings of 2014–2016 (Table S1) are presented as the average numbers and percentages of sea urchins per 6 h for the period of 60 h after their removal from the feeder. The average number of sea urchins for 24 h period before the removal is considered to be 100%. The data are extracted from Table S1. The experiments conducted on August 09, 2014, August 14, 2014 and August 20, 2016 were not included into statistical analysis because they might be influenced by the previous or next storm.

10.7717/peerj.8087/supp-10 Supplemental Information 10 Statistical parameters of unpaired *t*-test for differences in the numbers of sea urchin *Strongylocentrotus intermedius* before and after crushing the specimens of *Mesocentrotus nudus* near the feeders in long-term experiments.

10.7717/peerj.8087/supp-11 Supplemental Information 11 Changes in the numbers of sea urchins *Mesocentrotus nudus* in response to crushed conspecifics in short-term experiments. The data are presented as the numbers of sea urchins on the feeder and their percentages from the average number calculated as the mean of sea urchins which were present on the feeder during 21 h before the beginning of the treatment (indicated by red color). Nonlinear regression of data on the numbers of sea urchins during the period from 22 h (the moment of treatment) to 45 h was performed.

10.7717/peerj.8087/supp-12 Supplemental Information 12 Changes in the numbers of sea urchins *Mesocentrotus nudus* in response to crushed specimens of *Strongylocentrotus intermedius* in short-term experiments. The data are presented as the numbers of sea urchins on the feeder and their percentages from the average number calculated as the mean of sea urchins which were present on the feeder during 21 h before the beginning of the treatment (indicated by red color).

10.7717/peerj.8087/supp-13 Supplemental Information 13 Temporal dynamics of the mean group size of sea urchins *Mesocentrotus nudus* in response to crushed specimens of *Strongylocentrotus intermedius* in short-term experiments. The time of treatment is indicated by red color.

10.7717/peerj.8087/supp-14 Supplemental Information 14 Temporal dynamics of the mean group size of sea urchins *Strongylocentrotus intermedius* in response to crushed conspecifics in short-term experiments. The time of treatment is indicated by red color.

10.7717/peerj.8087/supp-15 Supplemental Information 15 Temporal dynamics of the numbers of sea urchins *Strongylocentrotus intermedius* in response to crushed conspecifics in short-term experiments. The data are presented as the numbers of sea urchins on the feeder and their percentages from the average number calculated as the mean of sea urchins which were present on the feeder during 21 h before the beginning of the treatment (indicated by red color).

10.7717/peerj.8087/supp-16 Supplemental Information 16 Movement activity of the sea urchins *Strongylocentrotus intermedius* in response to crushed conspecifics in short-term experiments. (A) The changes in the dinstances (cm) of 10 *S. intermedius* individuals from the place where conspecifics were crushed. (B) The changes in the step length (cm) of 10 *S. intermedius* individuals in response to crushed conspecifics.

10.7717/peerj.8087/supp-17 Supplemental Information 17 Temporal dynamics of the mean group size of sea urchins *Strongylocentrotus intermedius* in response to crushed specimens of *Mesocentrotus nudus* in short-term experiments. The time of treatment is indicated by red color.

10.7717/peerj.8087/supp-18 Supplemental Information 18 Temporal dynamics of the numbers of sea urchins *Strongylocentrotus intermedius* in response to crushed specimens of *Mesocentrotus nudus* in short-term experiments. The data are presented as the numbers of sea urchins on the feeder and their percentages from the average number calculated as the mean of sea urchins which were present on the feeder during 21 h before the beginning of the treatment (indicated by red color).

10.7717/peerj.8087/supp-19 Supplemental Information 19 Changes in the level sea related to wave activity in Kievka Bay before and during the storm of 9–12 September 2015 recorded by the depth sensor of a multi-parameter RBRXRX-620 datalogger. Green and violet lines indicate the time intervals (240 min) before the storm and in the beginning of the storm, respectively, when the distances traversed by 10 sea urchins of each species were tracked. *X*-axes: date and time of the day.

10.7717/peerj.8087/supp-20 Supplemental Information 20 Behavioral response of sea urchins *Strongylocentrotus intermedius* to starfish attack. (A) Video frame showing sea urchins relatively evenly distributed on the surface of the feeder with laminaria and an ailing sea urchin specimen (arrow). (B) Video frame showing several sea stars consuming an ailing sea urchin specimen (arrow) and several sea urchin groups (marked by white circles and ovals) on the surfaces and at the base of the stones surrounding the feeder. (C) Video frame showing that most sea urchins left the feeder and remains of the consumed specimen (arrow). (D) Video frame showing that sea urchins returned to the surface of the feeder.

10.7717/peerj.8087/supp-21 Supplemental Information 21 The changes in the locomotion speed of the sea urchins *Mesocentrotus nudus* and *Strongylocentrotus intermedius* in response to crushed conspecifics (A, B) and heterospecifics (C, D). Data are calculated from the data presented on Figs. 5, 7 and 10 and are presented as range (whiskers), upper and lower quartile (box), mean (+), and median (solid line). Different lowercase letters above the boxes indicate significant differences at *P* < 0.0001 (Kruskal–Wallis test followed by Dunn’s multiple comparisons test). *X*-axes: time intervals before (the first box plot) and after treatment. The last box plot represents the average speed for the entire period of measurements.

10.7717/peerj.8087/supp-22 Supplemental Information 22 The numbers of sea urchins *Mesocentrotus nudus* before and after the experiments with crushed conspecifics followed by the mimicking the conditions of stormy weather. The experiments were ****conducted during long-term recordings of 2014 and 2016 (Figs. 1A–1C; Table S1). Range (whiskers), upper and lower quartile (box), mean (+), and median (solid line) of the numbers of sea urchins before, during and after the storm periods are presented. Lowercase letters ‘*a’* above the boxes indicate significant differences in sea urchin numbers (Mann–Whitney test, *P* = 0.0007 and *P* = 0.0002 in 2014 and 2016, respectively).

10.7717/peerj.8087/supp-23 Supplemental Information 23 Behavioral responses of sea urchins *Mesocentrotus nudus* in short-term experiments. (A) Changes in *M. nudus* numbers in response to crushed conspecifics. Nonlinear regression of data on the numbers of *M. nudus* after crushing of conspecifics (the period from 22 h to 45 h) is significant (*R^2^* = 0.9791, see Table S11 for statistics). (B) Changes in *M. nudus* ****numbers in response to crushed *S. intermedius* individuals. Data are presented as median and range. Linear regression is not significant (*P* = 0.1639, see Table S12 for statistics). (C) Changes in the mean group size of *M. nudus* ****in response to crushed *S. intermedius* individuals are not significant (see Table S13 for statistics). The number of sea urchins (A) and mean group size (C) are presented as box-whisker plots showing the medians (solid lines), range (whiskers) and upper and lower quartiles (boxes). Upside down triangles denote the moments when sea urchins were crushed near the feeder.

## Additional Information and Declarations

### Data Availability

The following information was supplied regarding data availability: The raw data are available in the Supplemental Files.
