# Supplementary material for: Long-term study of behaviors of two cohabiting sea urchin species, Mesocentrotus nudus and Strongylocentrotus intermedius, under conditions of high food quantity and predation risk in situ
Source: PeerJ. 2019 Nov 22;7:e8087. doi: 10.7717/peerj.8087 (PMC6876488; doi:10.7717/peerj.8087)
Supplement: Supplemental Information 7 — Data are presented as mean ± SEM (n = 10) and the range (in the parentheses) for 240 min interval. [file peerj-07-8087-s007.docx]

| **Parameter** | ***M. nudus*** | ***S. intermedius*** | **Statistics** |
| --- | --- | --- | --- |
| Number of steps |  |  |  |
| Calm weather | 110 ± 12  (50–160) | 160 ± 10  (110–211) | *t*_10_ = 3.206; df=18; *p* = 0.0049 |
| Stormy weather | 21 ± 3  (9–35) | 57 ± 12  (18–141) | *t*_10_ = = 2.898; df = 9.868; *p* = 0.0161 |
| Step length, cm |  |  |  |
| Calm weather | 0.71 ± 0.08  (0.10–9.54) | 0.28 ± 0.03  (0.10–1.34) | *t*_10_ = 4.938; df=11.46; p = 0.0004 |
| Stormy weather | 0.29 ± 0.02  (0.10–1.34) | 0.33 ± 0.08  (0.10–4.14) | *t*_10_ = 0.5117; df=10.47; *p* = 0.6195 |
| Number of moves |  |  |  |
| Calm weather | 28 ± 2  (22–35) | 31 ± 2  (16–39) | *t*_10_ = 1.105; df=18; *p* = 0.2836 |
| Stormy weather | 13 ± 2  (5–23) | 26 ± 3  (13–39) | *t*_10_ = 3.569; df = 14.79; *p* = 0.0029 |
| Move length, cm |  |  |  |
| Calm weather | 2.99 ± 0.58  (0.10–56.5) | 1.65 ± 0.35  (0.10–39.72) | Mann-Witney U = 14.56; *p* = 0.0056 |
| Stormy weather | 0.57 ± 0.16  (0.10–8.37) | 0.65 ± 0.17  (0.10–37.7) | Mann-Witney U = 49; *p* = 0.95 |
| Entire distance traversed, cm |  |  |  |
| Calm weather | 76.90 ± 11.86  (34.91–146.40) | 44.28 ± 5.56  (29.73–88.85) | *t*_10_ = 2.491; df = 12.77; *p* = 0.0273 |
| Stormy weather | 5.87 ± 0.7  (3.91–9.97) | 16.10 ± 4.3  (3.80–45.76) | *t*_10_ = 2.346; df = 9.475; *p* = 0.0422 |
| Number of stops |  |  |  |
| Calm weather | 28 ± 2  (22–35) | 31 ± 2  (18–39) | *t*_10_ = 1.384; df = 18; *p* = 0.1833 |
| Stormy weather | 14 ± 2  (5–24) | 26 ± 3  (12–38) | t_10_ = 3.626; df=18; p = 0.0019 |
| Stop duration, min |  |  |  |
| Calm weather | 4.82 ± 0.54  (1–43) | 2.54 ± 0.26  (1–29) | *t*_10_ = 3.804; df = 18; *p* = 0.0013 |
| Stormy weather | 19.69 ± 3.02  (1–87) | 8.63 ± 1.70  (1–80) | *t*_10_ = 3.191; df = 18; *p* = 0.0051 |
| Entire stop duration, min |  |  |  |
| Calm weather | 130.0 ± 11.87  (80–190) | 80.1 ± 10.1  (29–130) | *t*_10_ = 3.206; df = 18; *p* = 0.0049 |
| Stormy weather | 217.9 ± 2.73  (205–231) | 183.2 ± 11.97  (99–222) | *t*_10_ = 2.827; df = 9.935; *p* = 0.018 |
| Speed, cm min^–1^ |  |  |  |
| Calm weather | 0.32 ± 0.05  (0.15–0.61) | 0.19 ± 0.02  (0.12–0.37) | Mann-Witney U = 17.5; *p* = 0.0123 |
| Stormy weather | 0.03 ± 0.003  (0.02–0.04) | 0.07 ± 0.02  (0.02–0.19) | Mann-Witney U = 11; *p* = 0.0018 |
