# Supplementary material for: Long-term study of behaviors of two cohabiting sea urchin species, Mesocentrotus nudus and Strongylocentrotus intermedius, under conditions of high food quantity and predation risk in situ
Source: PeerJ. 2019 Nov 22;7:e8087. doi: 10.7717/peerj.8087 (PMC6876488; doi:10.7717/peerj.8087)
Supplement: Supplemental Information 10 [file peerj-07-8087-s010.docx]

| **Experiment No.** | **Before/After** | **Mean** | **SEM** | **N** | ***t*** | **df** | ***p*** |
| --- | --- | --- | --- | --- | --- | --- | --- |
| Experiment 1 (August 28, 2014) | Before | 57 | 3.08 | 4 |  |  |  |
|  | After | 67 | 1.14 | 27 | 3.21 | 29 | 0.0032 |
| Experiment 2 (September 08, 2014) | Before | 65 | 0.65 | 4 |  |  |  |
|  | After | 70 | 0.62 | 22 | 3.64 | 24 | 0.0013 |
| Experiment 4 (August 26, 2016) | Before | 51 | 2.02 | 4 |  |  |  |
|  | After | 58 | 0.89 | 55 | 2.09 | 57 | 0.0416 |
