# Supplementary figures and images for: Long-term study of behaviors of two cohabiting sea urchin species, Mesocentrotus nudus and Strongylocentrotus intermedius, under conditions of high food quantity and predation risk in situ
Source: PeerJ. 2019 Nov 22;7:e8087. doi: 10.7717/peerj.8087 (PMC6876488; doi:10.7717/peerj.8087)

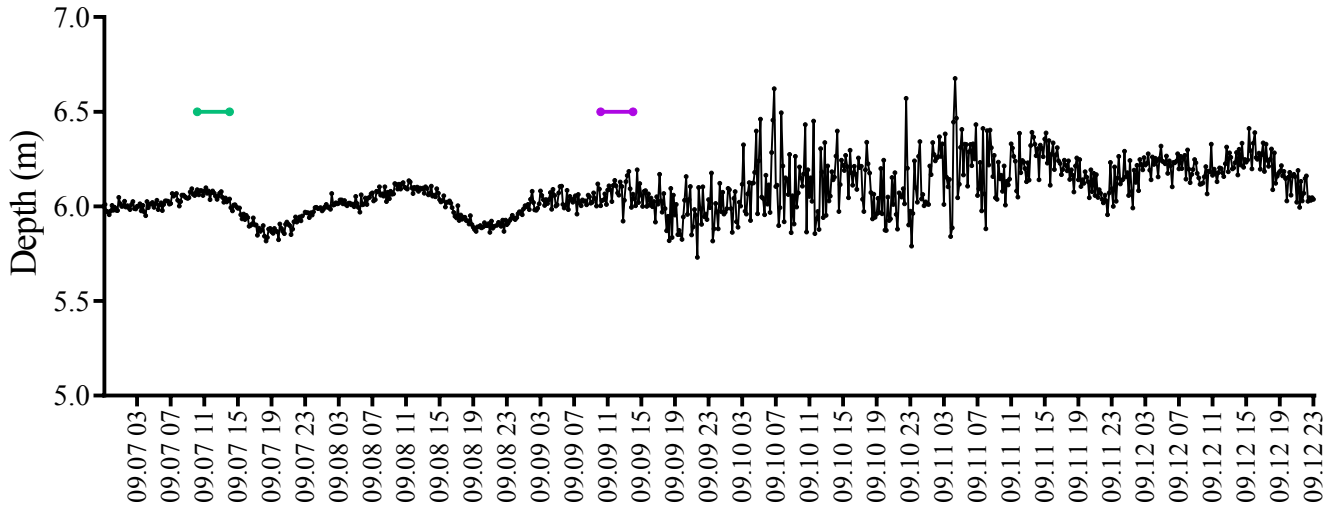

Supplement: Supplemental Information 19 — Green and violet lines indicate the time intervals (240 min) before the storm and in the beginning of the storm, respectively, when the distances traversed by 10 sea urchins of each species were tracked. X-axes: date and time of the day. [file peerj-07-8087-s019.pdf]

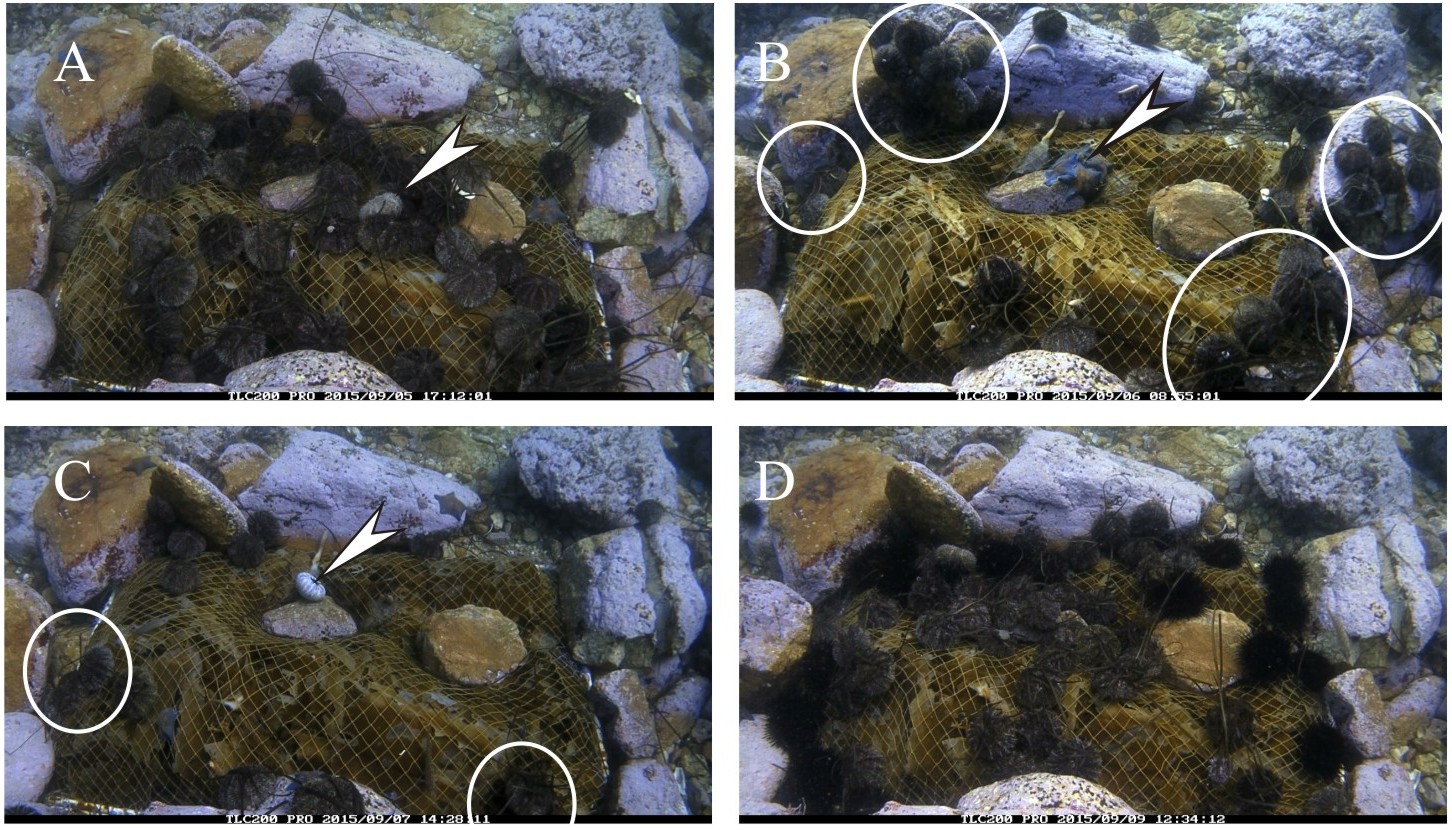

Supplement: Supplemental Information 20 — (A) Video frame showing sea urchins relatively evenly distributed on the surface of the feeder with laminaria and an ailing sea urchin specimen (arrow). (B) Video frame showing several sea stars consuming an ailing sea urchin specimen (arrow) and several sea urchin groups (marked by white circles and ovals) on the surfaces and at the base of the stones surrounding the feeder. (C) Video frame showing that most sea urchins left the feeder and remains of the consumed specimen (arrow). (D) Video frame showing that sea urchins returned to the surface of the feeder. [file peerj-07-8087-s020.jpg]

*Mesocentrotus nudus*

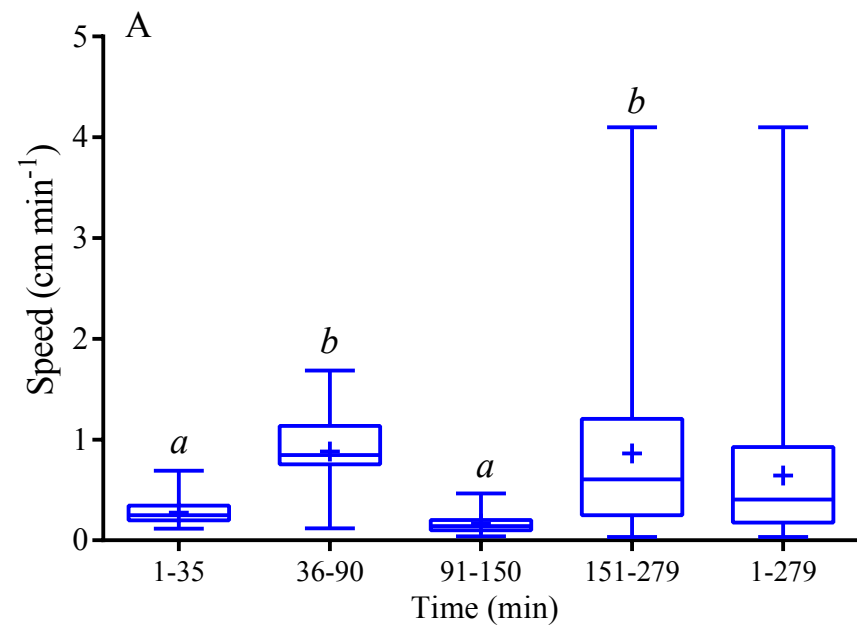

*Strongylocentrotus intermedius*

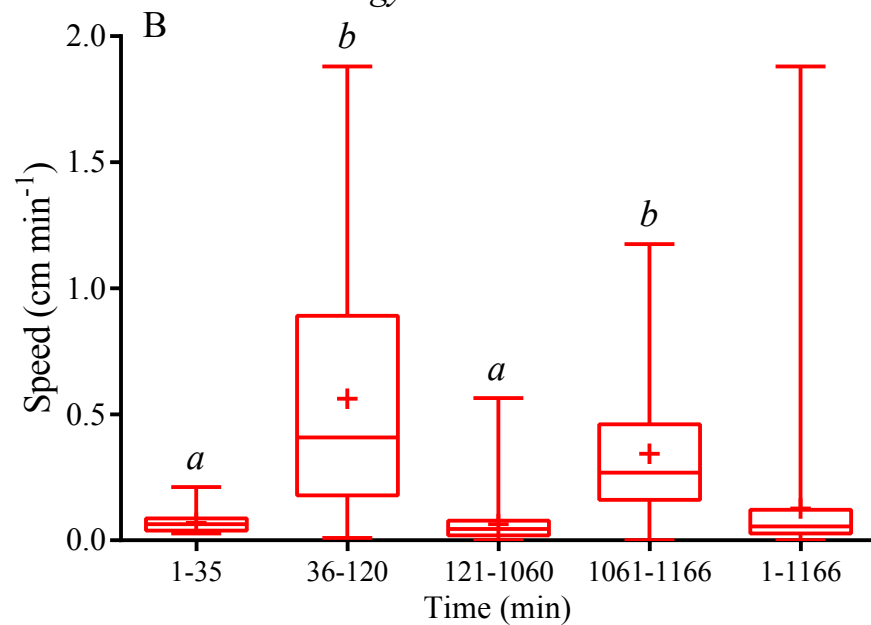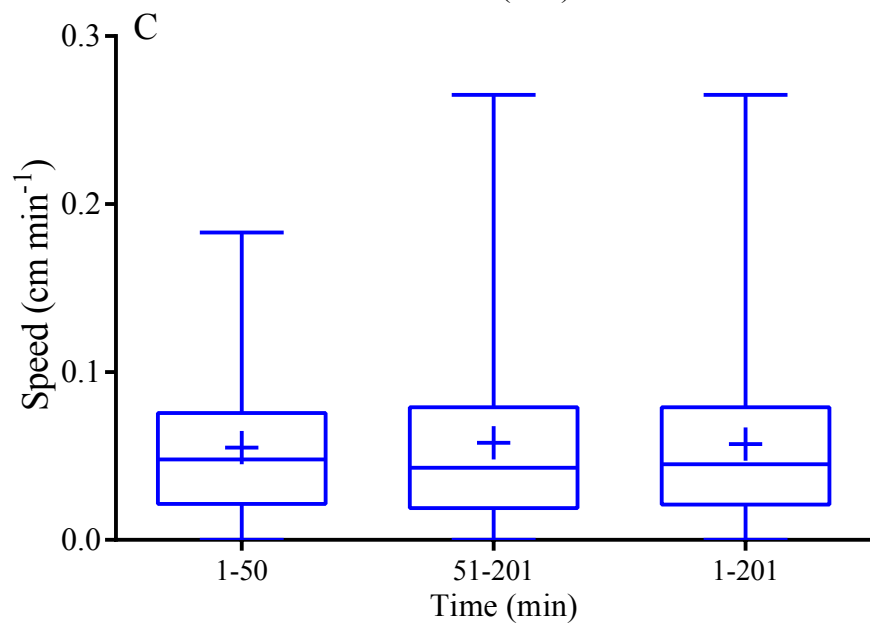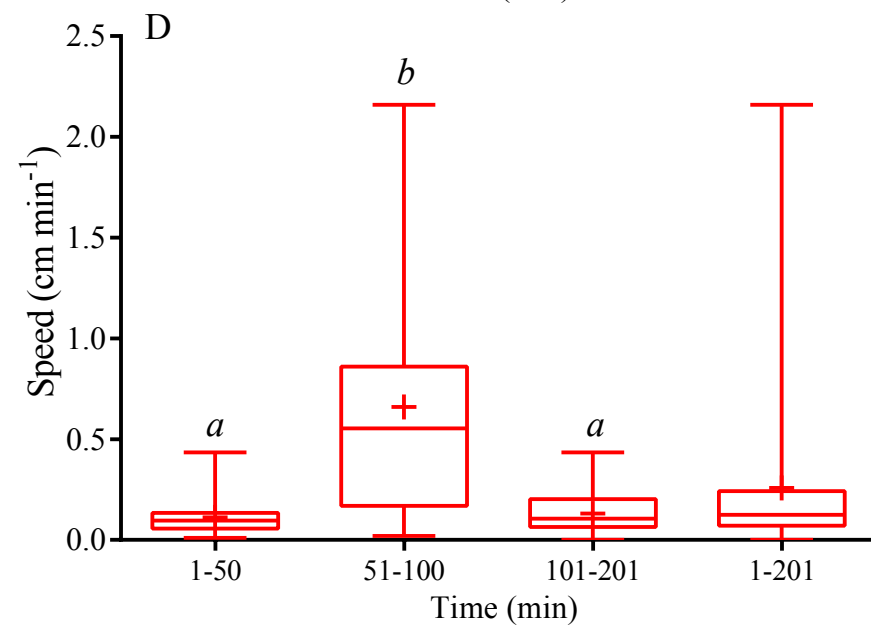

Supplement: Supplemental Information 21 — Data are calculated from the data presented on Figs. 5, 7 and 10 and are presented as range (whiskers), upper and lower quartile (box), mean (+), and median (solid line). Different lowercase letters above the boxes indicate significant differences at P < 0.0001 (Kruskal–Wallis test followed by Dunn’s multiple comparisons test). X-axes: time intervals before (the first box plot) and after treatment. The last box plot represents the average speed for the entire period of measurements. [file peerj-07-8087-s021.pdf]

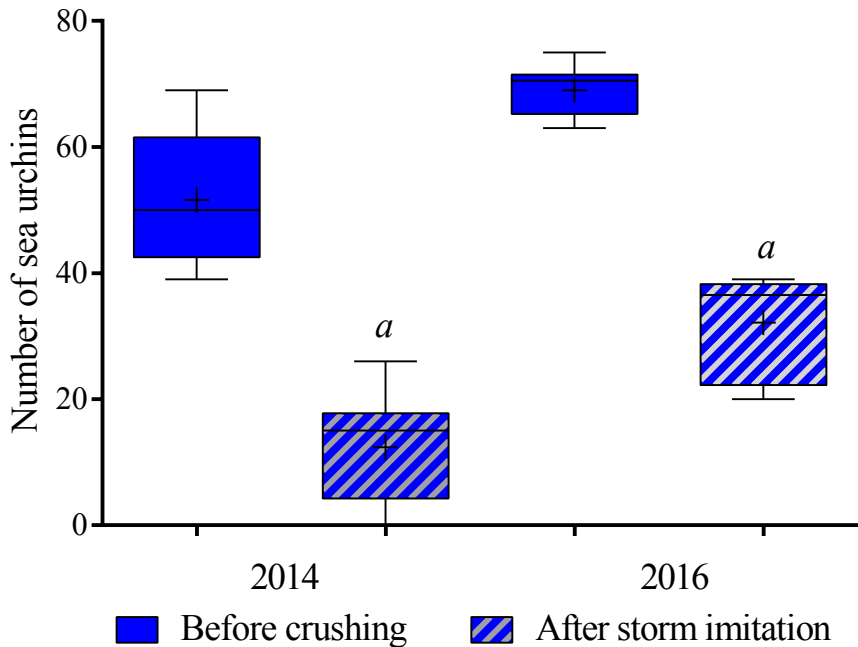

Supplement: Supplemental Information 22 — The experiments were conducted during long-term recordings of 2014 and 2016 (Figs. 1A–1C; Table S1). Range (whiskers), upper and lower quartile (box), mean (+), and median (solid line) of the numbers of sea urchins before, during and after the storm periods are presented. Lowercase letters ‘a’ above the boxes indicate significant differences in sea urchin numbers (Mann–Whitney test, P = 0.0007 and P = 0.0002 in 2014 and 2016, respectively). [file peerj-07-8087-s022.pdf]

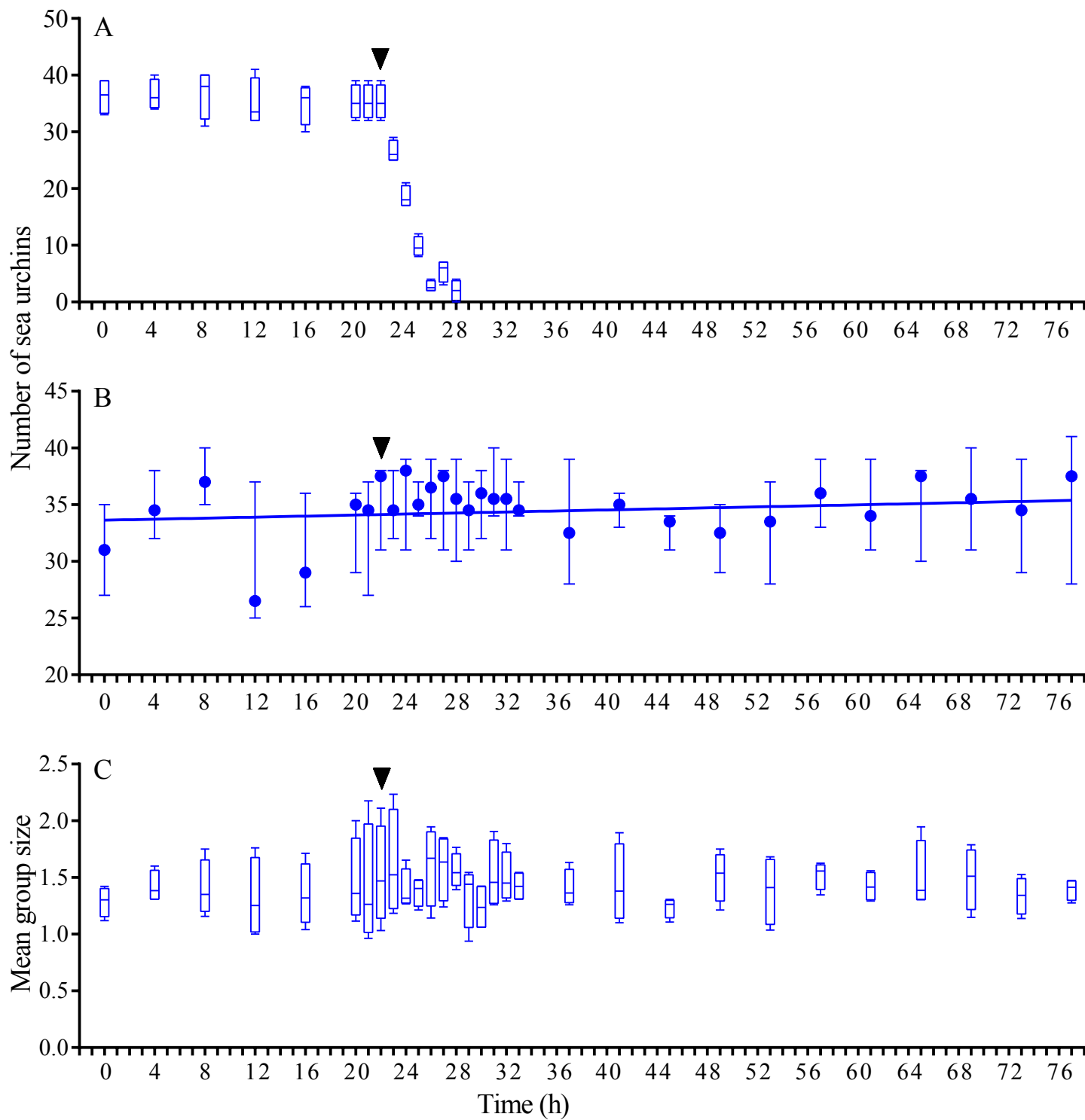

Supplement: Supplemental Information 23 — (A) Changes in M. nudus numbers in response to crushed conspecifics. Nonlinear regression of data on the numbers of M. nudus after crushing of conspecifics (the period from 22 h to 45 h) is significant (R2 = 0.9791, see Table S11 for statistics). (B) Changes in M. nudus numbers in response to crushed S. intermedius individuals. Data are presented as median and range. Linear regression is not significant (P = 0.1639, see Table S12 for statistics). (C) Changes in the mean group size of M. nudus in response to crushed S. intermedius individuals are not significant (see Table S13 for statistics). The number of sea urchins (A) and mean group size (C) are presented as box-whisker plots showing the medians (solid lines), range (whiskers) and upper and lower quartiles (boxes). Upside down triangles denote the moments when sea urchins were crushed near the feeder. [file peerj-07-8087-s023.pdf]
